# Socio-Economic Position and Type 2 Diabetes Risk Factors: Patterns in UK Children of South Asian, Black African-Caribbean and White European Origin

**DOI:** 10.1371/journal.pone.0032619

**Published:** 2012-03-07

**Authors:** Claudia Thomas, Claire M. Nightingale, Angela S. Donin, Alicja R. Rudnicka, Christopher G. Owen, Naveed Sattar, Derek G. Cook, Peter H. Whincup

**Affiliations:** 1 Population Health Research Centre, Division of Population Health Sciences and Education, St Georges, University of London, London, United Kingdom; 2 BHF Glasgow Cardiovascular Research Centre, University of Glasgow, Glasgow, United Kingdom; German Diabetes Center, Leibniz Center for Diabetes Research at Heinrich Heine University Duesseldorf, Germany

## Abstract

**Background:**

Socio-economic position (SEP) and ethnicity influence type 2 diabetes mellitus (T2DM) risk in adults. However, the influence of SEP on emerging T2DM risks in different ethnic groups and the contribution of SEP to ethnic differences in T2DM risk in young people have been little studied. We examined the relationships between SEP and T2DM risk factors in UK children of South Asian, black African-Caribbean and white European origin, using the official UK National Statistics Socio-economic Classification (NS-SEC) and assessed the extent to which NS-SEC explained ethnic differences in T2DM risk factors.

**Methods and Findings:**

Cross-sectional school-based study of 4,804 UK children aged 9–10 years, including anthropometry and fasting blood analytes (response rates 70%, 68% and 58% for schools, individuals and blood measurements). Assessment of SEP was based on parental occupation defined using NS-SEC and ethnicity on parental self-report. Associations between NS-SEC and adiposity, insulin resistance (IR) and triglyceride differed between ethnic groups. In white Europeans, lower NS-SEC status was related to higher ponderal index (PI), fat mass index, IR and triglyceride (increases per NS-SEC decrement [95%CI] were 1.71% [0.75, 2.68], 4.32% [1.24, 7.48], 5.69% [2.01, 9.51] and 3.17% [0.96, 5.42], respectively). In black African-Caribbeans, lower NS-SEC was associated with lower PI (−1.12%; [−2.01, −0.21]), IR and triglyceride, while in South Asians there were no consistent associations between NS-SEC and T2DM risk factors. Adjustment for NS-SEC did not appear to explain ethnic differences in T2DM risk factors, which were particularly marked in high NS-SEC groups.

**Conclusions:**

SEP is associated with T2DM risk factors in children but patterns of association differ by ethnic groups. Consequently, ethnic differences (which tend to be largest in affluent socio-economic groups) are not explained by NS-SEC. This suggests that strategies aimed at reducing social inequalities in T2DM risk are unlikely to reduce emerging ethnic differences in T2DM risk.

## Introduction

Type 2 diabetes mellitus (T2DM) is a major emerging public health problem, both in the UK and worldwide [1]. T2DM and its key risk factors (particularly obesity) show marked ethnic differences and associations with socio-economic position (SEP). In the UK, the risks of obesity and T2DM are markedly higher among South Asians (including those of Indian, Pakistani and Bangladeshi origin) and moderately higher among black African-Caribbeans (including both African and Caribbean origins) compared to white Europeans [2]. Among white Europeans, low SEP is associated with higher risks of obesity and T2DM [2]–[5], a pattern reported in many higher-income populations [6], [7]. However, there are few data on the associations of SEP and T2DM and its risk factors among ethnic minority groups, though recent reports have suggested that in adults similar socio-economic gradients (low SEP associated with higher risks of obesity and T2DM) may be emerging both among South Asians [2], [8] and among black African-Caribbeans [2]. Although it has been suggested that ethnic differences in cardiovascular disease and T2DM could be explained by ethnic differences in SEP [8], [9], few studies have investigated this issue directly.

T2DM has its origins in early life [10], [11] and is becoming increasingly common in childhood and adolescence, particularly among South Asians and other ethnic minority groups [12]. Population-wide ethnic differences in blood glucose, markers of insulin resistance and adiposity have been reported both in UK adolescents and children [13]–[16]. Although socio-economic patterns of adiposity and T2DM risk markers have been examined in white European children [17], [18], little is known about the influence of SEP onT2DM and its risk markers (particularly hyperglycaemia, insulin resistance, obesity and dyslipidaemia) in children of different ethnic origins. Moreover, the extent to which socio-economic differences account for emerging ethnic differences in T2DM risk has been little studied.

The primary aim of this paper was to examine the relationships between SEP and T2DM risk factors (including markers of the early emergence of T2DM risk [particularly insulin resistance, blood glucose and triglyceride] and underlying determinants of T2DM [particularly adiposity]) in UK children of South Asian, black African-Caribbean and white European origin. We examined patterns both in all South Asians and in all black African-Caribbeans together, and then separately in Indian, Pakistani, Bangladeshi, black African and black Caribbean groups. Analyses were carried out using the National Statistics Socio-economic Classification (NS-SEC), the official UK socioeconomic classification, examined both as a hierarchical (ordered) and non-hierarchical classification and including economically inactive individuals as a separate group. A second aim was to assess the extent to which NS-SEC may explain previously described ethnic differences in emerging T2DM risk factors [15].

## Methods

### Ethics statement

The study was approved by the Multicentre Research Ethics Committee (Wales). Informed written consent was obtained from each pupil's parent or guardian.

### Study design

The Child Heart and Health Study in England (CHASE) is an investigation of the cardiovascular health of British school children aged 9–10years of white European, South Asian and black African-Caribbean origin. Full details of the study design have been reported elsewhere [15], [19]. In brief, the study took place in 200 primary schools in London, Leicester and Birmingham, sampled to include 100 schools with a high proportion (20–80%) of South Asian pupils and 100 schools with a high proportion of black African-Caribbean pupils. All Head Teachers were approached by the Principal Investigator and invited to participate; 140 (70%) agreed. Non-participating schools were replaced by a school from the sampling frame with a similar ethnic mix and in the same or a neighbouring borough. The final sample included 183 schools from London, 14 from Birmingham and 3 from Leicester.

### Measurements

Assessments were carried out during school terms by a single research team visiting schools in different areas in rotation. Participating children had physical measurements (anthropometry, blood pressure and spirometry) and provided a fasting blood sample. Height was measured to the last complete millimetre with a portable stadiometer (Chasmors Ltd, London, UK) and weight with an electronic digital scale (Tanita Inc, Tokyo, Japan). Ponderal index was calculated as kg/m^3^. Waist circumference was measured at the midpoint between the lower margin of the ribs and the iliac crest in the mid-axillary line. Right-sided skinfolds (biceps, triceps, subscapular, suprailiac) were measured and summed for analysis. Body composition was measured using leg to arm bioimpedance (Bodystat 1500 bioimpedance monitor, Bodystat Ltd, Isle of Man, UK). Fat mass was calculated using equations derived for children using dual-emission X-ray absorptiometry [20], and presented as fat mass index independent of height (fat mass/m^5^).

Blood samples were transferred for analysis of HbA1c, glucose, and blood lipids within 48 hours of collection. Glucose was measured in plasma using the hexokinase method. HbA1c was measured in whole blood by ion exchange high performance liquid chromatography and adjusted for abnormal haemoglobin variants or for increased amounts of normal variant fetal haemoglobin where present. Serum triglyceride and HDL-cholesterol were measured using an Olympus auto-analyser. Serum, separated and frozen on dry ice after collection, was measured for insulin using an ELISA method and for C-reactive protein (CRP) using ultra-sensitive nephelometry (Dade Behring, Milton Keynes, UK). The homeostasis model assessment (HOMA) equations were used to provide an estimate of insulin resistance [21].

Parents completed a questionnaire on their self-defined ethnicity, ethnicity of the participating child and their occupations. Ethnicity of the children was defined using the ethnicity of both parents or (if not available) the ethnicity of the child or (if not available) place of birth of parents and grandparents provided by the child (1%). In the present analyses ‘white European’ includes children whose ethnic origin was defined as ‘white British’ ‘white Irish’ and ‘white European’ (or a combination of these). ‘South Asian’ includes ‘Indian’ ‘Pakistani’ ‘Bangladeshi’ and ‘Sri Lankan’ (or a combination of these). ‘Other Asian’ includes ‘Asian other’ and ‘other’ with a specified Asian place of origin (mainly Afghanistan, China and Turkey). ‘Black African-Caribbean’ includes ‘black African’ ‘black Caribbean’ ‘black British’ and ‘black other’ (or a combination of these). The ‘other’ ethnic group includes all other categories of individual and mixed ethnic origins. The ethnic subcategories ‘Indian’ ‘Pakistani’ ‘Bangladeshi’ includes children whose parents both originated in the same country; ‘black African’ and ‘black Caribbean’ groups those who originated in the same region. For primary analyses, all children of South Asian origin and all black children originally of African origin have been considered together; in a second level of analysis SEP patterns have been examined separately in children of Indian, Pakistani and Bangladeshi origin and separately in black children of African and Caribbean origin.

Current socio-economic position (SEP) was measured according to the UK National Statistics Socioeconomic Classification (NS-SEC), coded from parental occupation (Standard Occupational Classification 2000) using an Office for National Statistics matrix [22]. The SEP of the child was defined on the basis of the parent with the highest NS-SEC grade (or that of the sole parent in single parent households, 25%). NS-SEC was operationalized using both the three class version (NS-SEC3 categorised as professional and managerial, intermediate, routine and manual) and the five class version (NS-SEC5 categorised as managerial and professional, intermediate, small employers and own account workers, lower supervisory and technical, semi-routine and routine). An additional category of ‘economically inactive’ was added at the lower end of both NS-SEC versions, in accordance with published guidance on NS-SEC use [22]. Individuals who could not be classified into an NS-SEC group are shown in Tables as ‘unclassified’ but have not been included in statistical analyses.

### Statistical analysis

All analyses were carried out in STATA (version 11.1; StataCorp LP, College Station, TX, USA). Multilevel linear regression models fitting school as a random effect in order to take account of the natural clustering of children within school were used to provide adjusted means and adjusted differences in risk factors and their 95% confidence intervals. Analyses were adjusted for sex, age, month of assessment and observer (physical measurements only) as fixed effects. All outcome variables were log transformed and ethnic and socio-economic differences in these variables were expressed as percentage change (exp(ß)-1)*100 in order to achieve normality and to enable comparisons across outcomes.

Associations between NS-SEC3 and T2DM risk factors have treated NS-SEC3 both as an ordinal variable and as a nominal variable [22]. Associations between NS-SEC5 and T2DM risk factors have treated NS-SEC5 entirely as a nominal variable. The principal analyses have included ‘economically inactive’ individuals, treating them as the most deprived group in analyses of trends across ordinal categories; sensitivity analyses have been carried out excluding the economically inactive groups and therefore restricted to currently employed groups alone.

We examined the influence of NS-SEC on ethnic differences on T2DM risk factors by (i) adjusting ethnic differences for NS-SEC5, and (ii) where there was evidence of interaction (for markers of adiposity and insulin resistance in particular and more weakly for triglyceride levels) by examining the ethnic differences stratified by NS-SEC3 groups. We also investigated the effect of adjustment for parental education and an index of household amenities (data not presented) in order to address the potential for residual confounding by other dimensions of SEP.

Tests of interaction between ethnic group and NS-SEC and between gender and NS-SEC were carried out using the likelihood ratio (LR) test; these tests were carried out with NS-SEC fitted both as ordinal and nominal variables. The associations between NS-SEC and T2DM risk factors were similar in boys and girls, both overall and within specific ethnic groups, and data are therefore presented for both groups combined, adjusted for sex. Analyses were undertaken for the main ethnic groups and repeated using ethnic sub-groups.

## Results

### Descriptive characteristics

Of 8,641 children invited, 5,887 (68%) participated and 4843 (82%) children without type 1 diabetes provided fasting blood samples. Analyses were based on 4804 children (1158 white European, 1201 African-Caribbean, 1314 South Asian, 295 other Asian children and 836 of other ethnic origins) with complete data on parental employment status. The mean age of participants was 10.0 years (s.d. = 0.4 years) and 51% were female. Participation rates were unrelated to age but were slightly lower among males (65%) than females (71%). Participation rates were similar among white Europeans, South Asians, other Asians and other ethnic groups (69%, 73%, 70% and 71% respectively) with slightly lower participation among black African-Caribbeans (65%). The socioeconomic status of children who did and did not provide blood samples did not differ appreciably (both groups included 27% managerial/professional and 17% economically inactive).

There were marked differences in the distribution of NS-SEC categories between the main ethnic groups (Table 1). White European and black African-Caribbean children had higher proportions of parents in managerial/professional occupations and lower proportions in routine/manual occupations and economically inactive than South Asian and other Asian children. Within the main ethnic groupings, Pakistani and Bangladeshi children were slightly more disadvantaged than Indian children and black African children more so than black Caribbean children.

**Table 1:** Socio-economic position (NS-SEC) by ethnic group in CHASE, n (%).

	White European	Black African-Caribbean			South Asian				Other Asian	Other	All CHASE
		Caribbean	African	Other	Indian	Pakistani	Bangladeshi	Other			
**I. Managerial & professional**	358 (30.9)	148 (32.2)	193 (29.4)	27 (31.8)	121 (29.7)	87 (18.2)	36 (11.1)	23 (21.7)	40 (13.6)	266 (31.8)	1299 (27.0)
**II. Intermediate**											
Intermediate	155 (13.4)	83 (18.0)	67 (10.2)	9 (10.6)	86 (21.1)	44 (9.2)	10 (3.1)	14 (13.2)	31 (10.5)	95 (11.4)	594 (12.4)
Small employers & own account	173 (14.9)	51 (11.1)	36 (5.5)	6 (7.1)	49 (12.0)	83 (17.4)	32 (9.9)	6 (5.7)	42 (14.2)	87 (10.4)	565 (11.8)
**III. Routine & Manual**											
Lower supervisory & technical	65 (5.6)	24 (5.2)	16 (2.4)	2 (2.3)	17 (4.2)	17 (3.6)	3 (0.9)	3 (2.8)	11 (3.7)	27 (3.2)	185 (3.9)
Semi-routine & routine	213 (18.4)	90 (19.6)	141 (21.5)	20 (23.5)	90 (22.1)	123 (25.8)	121 (37.5)	44 (41.5)	85 (28.8)	186 (22.2)	1113 (23.2)
**IV. Economically inactive**	163 (14.1)	35 (7.6)	136 (20.7)	16 (18.8)	29 (7.1)	100 (21.0)	108 (33.4)	16 (15.1)	70 (23.7)	131 (15.7)	804 (16.7)
**V. Unclassifiable**	31 (2.7)	29 (6.3)	67 (10.2)	5 (5.9)	16 (3.9)	23 (4.8)	13 (4.0)	0 (0.0)	16 (5.4)	44 (5.3)	244 (5.1)
Total	1158 (100)	460 (100)	656 (99.9)	85 (100)	408 (100.1)	477 (100)	323 (99.9)	106 (100)	295 (99.9)	836 (100)	4804 (100.1)

Some column percentages do not total 100 due to rounding errors.

### NS-SEC and type 2 diabetes risk factors in main ethnic groups

Geometric mean T2DM risk factor levels for each NS-SEC3 category and the percentage change in risk factor levels per one NS-SEC group decrement are presented for the whole sample and separately by main ethnic group in Tables 2 to [Table pone-0032619-t003] [Table pone-0032619-t004] 5.

**Table 2:** Adjusted means and percentage differences in height, weight and ponderal index by NS-SEC and ethnic group.

Outcome & NS-SEC	White European (n = 1158)		Black African-Caribbean (n = 1201)		South Asian (n = 1314)		All CHASE (n = 4804)¶		Difference between WE, AC & SA groups	
	Mean (95% CI)	*P*	Mean (95% CI)	*P*	Mean (95% CI)	*P*	Mean (95% CI)	*P*	*P* ‡	*P* §
**Height (cm)**										
Managerial & professional	139.1 (138.5, 139.8)		143.3 (142.6, 144.0)		138.5 (137.7, 139.3)		140.3 (139.9, 140.6)			
Intermediate	139.4 (138.7, 140.1)		142.7 (141.9, 143.5)		138.6 (137.9, 139.3)		140.0 (139.6, 140.4)			
Routine & manual	138.4 (137.7, 139.2)		142.9 (142.1, 143.7)		138.7 (138.0, 139.3)		139.8 (139.4, 140.1)			
Economically inactive	139.0 (138.0, 140.0)		141.5 (140.6, 142.5)		138.2 (137.4, 139.0)		139.5 (139.1, 140.0)			
Unclassified	138.7 (136.4, 141.0)		144.2 (142.9, 145.6)		139.0 (137.2, 140.8)		140.5 (139.7, 141.3)			
% difference per NS-SEC†	−0.13 (−0.40, 0.14)	0.34	−0.33 (−0.59, −0.08)	0.01	−0.04 (−0.29, 0.22)	0.76	−0.17 (−0.30, −0.04)	0.01	0.22	
% difference per NS-SEC€	−0.22 (−0.60, 0.15)	0.24	−0.17 (−0.54, 0.20)	0.36	0.06 (−0.31, 0.42)	0.75	−0.16 (−0.35, 0.03)	0.08		
p-value NS-SEC (nominal)*		0.30		0.03		0.88		0.07		0.25
**Weight (kg)**										
Managerial & professional	34.7 (33.9, 35.5)		39.3 (38.4, 40.2)		33.9 (33.0, 34.9)		36.0 (35.6, 36.5)			
Intermediate	35.0 (34.2, 35.9)		39.1 (38.0, 40.2)		34.4 (33.5, 35.3)		36.0 (35.5, 36.5)			
Routine & manual	35.7 (34.8, 36.7)		39.1 (38.1, 40.2)		34.8 (34.0, 35.6)		36.3 (35.8, 36.8)			
Economically inactive	36.0 (34.7, 37.3)		36.1 (34.9, 37.3)		33.8 (32.9, 34.8)		35.5 (34.9, 36.1)			
Unclassified	34.4 (31.7, 37.3)		39.3 (37.6, 41.1)		34.8 (32.6, 37.0)		36.1 (35.0, 37.1)			
% difference per NS-SEC†	1.34 (0.01, 2.69)	0.04	−2.11 (−3.34, −0.85)	0.001	0.12 (−1.14, 1.40)	0.85	−0.26 (−0.91, 0.39)	0.42	<0.001	
% difference per NS-SEC€	1.44 (−0.41, 3.33)	0.12	−0.30 (−2.09, 1.52)	0.74	1.24 (−0.57, 3.08)	0.17	0.43 (−0.49, 1.36)	0.35		
p-value NS-SEC (nominal)*		0.25		<0.001		0.40		0.17		0.005
**Ponderal index (kg/m^3^)**										
Managerial & professional	12.9 (12.7, 13.1)		13.3 (13.1, 13.6)		12.8 (12.5, 13.0)		13.1 (12.9, 13.2)			
Intermediate	12.9 (12.7, 13.2)		13.5 (13.2, 13.7)		12.9 (12.7, 13.2)		13.1 (13.0, 13.3)			
Routine & manual	13.5 (13.2, 13.7)		13.4 (13.1, 13.7)		13.0 (12.8, 13.3)		13.3 (13.2, 13.4)			
Economically inactive	13.4 (13.1, 13.8)		12.7 (12.4, 13.0)		12.8 (12.5, 13.1)		13.1 (12.9, 13.2)			
Unclassified	12.9 (12.2, 13.7)		13.1 (12.7, 13.5)		12.9 (12.4, 13.5)		13.0 (12.7, 13.3)			
% difference per NS-SEC†	1.71 (0.75, 2.68)	<0.001	−1.12 (−2.01, −0.21)	0.01	0.20 (−0.71, 1.11)	0.66	0.24 (−0.23, 0.71)	0.31	<0.001	
% difference per NS-SEC€	2.08 (0.74, 3.44)	0.002	0.21 (−1.08, 1.52)	0.74	1.05 (−0.24, 2.36)	0.10	0.91 (0.24, 1.58)	0.01		
p-value NS-SEC (nominal)*		0.001		0.002		0.35		0.03		<0.001

Mean: means adjusted for sex, age, observer, month and school (random effect).

95% CI: 95% confidence interval of the mean.

¶ Estimates adjusted for ethnicity (all groups included).

‡ interaction test of NS-SEC and main ethnic groups (white European, black African-Caribbean, South Asian) and excluding “unclassified” NS-SEC group fitting NS-SEC as an ordinal variable.

§ interaction test of NS-SEC and main ethnic groups (white European, black African-Caribbean, South Asian) and excluding “unclassified” NS-SEC group fitting NS-SEC as a categorical variable.

† per NS-SEC decrement from professional to economically inactive (excluding unclassified group).

€ per NS-SEC decrement from professional to routine &manual (excluding economically inactive and unclassified groups).

* p-value for NS-SEC fitted as an unordered nominal variable (excluding unclassified group).

Note: Percentage variance due to school differences: height 0.3%, weight 0.9%, ponderal index 1.3%.

**Table 3 pone-0032619-t003:** Adjusted means and percentage differences in skinfolds, fat mass index and waist circumference by NSSEC and ethnic group.

Outcome & NS-SEC	White European (n = 1158)		Black African-Caribbean (n = 1201)		South Asian (n = 1314)		All CHASE (n = 4804)¶		Difference between WE, AC & SA groups	
	Mean (95% CI)	*P*	Mean (95% CI)	*P*	Mean (95% CI)	*P*	Mean (95% CI)	*P*	*P* ‡	*P* §
**Sum of skinfolds (mm)**										
Managerial & professional	38.4 (36.5, 40.4)		39.4 (37.5, 41.5)		41.9 (39.5, 44.4)		40.4 (39.3, 41.6)			
Intermediate	38.9 (36.9, 41.0)		40.1 (37.8, 42.6)		43.9 (41.6, 46.3)		41.0 (39.8, 42.2)			
Routine & manual	42.5 (40.1, 45.0)		41.2 (38.9, 43.5)		43.4 (41.4, 45.6)		42.0 (40.8, 43.2)			
Economically inactive	43.5 (40.4, 46.9)		38.7 (36.1, 41.4)		40.6 (38.2, 43.1)		40.6 (39.2, 42.0)			
Unclassified	36.5 (30.8, 43.2)		39.5 (35.9, 43.4)		42.6 (37.3, 48.5)		39.9 (37.5, 42.4)			
% difference per NS-SEC†	4.81 (1.95, 7.74)	0.001	0.10 (−2.51, 2.79)	0.94	−0.83 (−3.43, 1.84)	0.53	0.63 (−0.73, 2.02)	0.36	0.007	
% difference per NS-SEC€	5.10 (1.11, 9.24)	0.01	2.11 (−1.67, 6.04)	0.27	1.85 (−1.91, 5.76)	0.33	1.95 (−0.001, 3.94)	0.05		
p-value NS-SEC (nominal)*		0.005		0.52		0.19		0.21		0.06
**Fat mass index (kg/m^5^)**										
Managerial & professional	1.64 (1.55, 1.73)		1.81 (1.72, 1.92)		1.79 (1.68, 1.91)		1.75 (1.70, 1.81)			
Intermediate	1.63 (1.54, 1.73)		1.87 (1.76, 2.00)		1.88 (1.77, 1.99)		1.81 (1.75, 1.87)			
Routine & manual	1.84 (1.73, 1.96)		1.87 (1.76, 1.98)		1.91 (1.81, 2.01)		1.86 (1.80, 1.92)			
Economically inactive	1.79 (1.66, 1.94)		1.65 (1.53, 1.78)		1.78 (1.66, 1.90)		1.77 (1.70, 1.84)			
Unclassified	1.64 (1.37, 1.97)		1.77 (1.60, 1.96)		1.78 (1.55, 2.05)		1.76 (1.65, 1.88)			
% difference per NS-SEC†	4.32 (1.24, 7.48)	0.005	−1.96 (−4.72, 0.87)	0.16	0.26 (−2.59, 3.20)	0.86	0.96 (−0.53, 2.47)	0.20	0.007	
% difference per NS-SEC€	5.75 (1.46, 10.22)	0.01	1.41 (−2.59, 5.57)	0.49	3.20 (−0.87, 7.43)	0.12	3.03 (0.92, 5.19)	0.004		
p-value NS-SEC (nominal)*		0.01		0.05		0.25		0.03		0.04
**Waist circumference (cm)**										
Managerial & professional	63.1 (62.2, 64.0)		64.3 (63.4, 65.3)		62.5 (61.5, 63.6)		63.5 (63.0, 64.0)			
Intermediate	63.7 (62.7, 64.7)		64.4 (63.3, 65.5)		63.2 (62.2, 64.2)		63.8 (63.2, 64.3)			
Routine & manual	64.9 (63.9, 66.0)		64.6 (63.6, 65.7)		63.8 (62.9, 64.7)		64.4 (63.9, 64.9)			
Economically inactive	65.2 (63.8, 66.6)		62.7 (61.5, 64.0)		62.4 (61.3, 63.5)		63.6 (63.0, 64.3)			
Unclassified	63.3 (60.2, 66.5)		64.1 (62.4, 65.9)		63.0 (60.7, 65.5)		63.5 (62.4, 64.7)			
% difference per NS-SEC†	1.24 (0.42, 2.05)	0.002	−0.57 (−1.33, 0.20)	0.14	0.12 (−0.65, 0.89)	0.76	0.23 (−0.17, 0.63)	0.24	0.004	
% difference per NS-SEC€	1.41 (0.28, 2.56)	0.01	0.16 (−0.94, 1.27)	0.77	1.04 (−0.06, 2.16)	0.06	0.68 (0.12, 1.25)	0.02		
p-value NS-SEC (nominal)*		0.02		0.12		0.15		0.09		0.06

Mean: means adjusted for sex, age, observer, month and school (random effect). Missing values: sum of skinfolds (n = 12), fat mass index (n = 64), waist circumference (n = 1).

95% CI: 95% confidence interval of the mean.

¶ Estimates adjusted for ethnicity (all groups included).

‡ interaction test of NS-SEC and main ethnic groups (white European, black African-Caribbean, South Asian) and excluding “unclassified” NS-SEC group fitting NS-SEC as an ordinal variable.

§ interaction test of NS-SEC and main ethnic groups (white European, black African-Caribbean, South Asian) and excluding “unclassified” NS-SEC group fitting NS-SEC as a categorical variable.

† per NS-SEC decrement from professional to economically inactive (excluding unclassified group).

€ per NS-SEC decrement from professional to routine &manual (excluding economically inactive and unclassified groups).

* p-value for NS-SEC fitted as an unordered nominal variable (excluding unclassified group).

Note: Percentage variance due to school differences: sum of skinfolds 1.5%, fat mas index 3.5%, waist circumference 0.9%.

**Table 4 pone-0032619-t004:** Adjusted means and mean differences in HbA1c, glucose and insulin resistance by NS-SEC and ethnic group.

Outcome & NS-SEC	White European (n = 1158)		Black African-Caribbean (n = 1201)		South Asian (n = 1314)		All CHASE (n = 4804)¶		Difference between WE, AC & SA groups	
	Mean (95% CI)	*P*	Mean (95% CI)	*P*	Mean (95% CI)	*P*	Mean (95% CI)	*P*	*P* ‡	*P* §
**HbA1c (%)**										
Managerial & professional	5.16 (5.13, 5.20)		5.28 (5.24, 5.31)		5.27 (5.23, 5.31)		5.23 (5.21, 5.25)			
Intermediate	5.18 (5.15, 5.22)		5.24 (5.20, 5.28)		5.30 (5.27, 5.34)		5.25 (5.22, 5.27)			
Routine & manual	5.18 (5.14, 5.22)		5.29 (5.25, 5.32)		5.28 (5.25, 5.32)		5.24 (5.22, 5.26)			
Economically inactive	5.19 (5.14, 5.24)		5.28 (5.23, 5.33)		5.25 (5.21, 5.29)		5.24 (5.21, 5.26)			
Unclassified	5.17 (5.06, 5.28)		5.23 (5.17, 5.29)		5.24 (5.15, 5.33)		5.22 (5.17, 5.26)			
% difference per NSSEC†	0.18 (−0.17, 0.54)	0.30	0.06 (−0.28, 0.39)	0.74	−0.16 (−0.49, 0.18)	0.36	0.04 (−0.14, 0.22)	0.66	0.38	
% difference per NS-SEC€	0.21 (−0.28, 0.69)	0.40	−0.01 (−0.48, 0.47)	0.97	0.10 (−0.37, 0.57)	0.68	0.07 (−0.18, 0.31)	0.59		
p-value NS-SEC (nominal)*		0.86		0.42		0.25		0.73		0.28
**Glucose (mmol/L)**										
Managerial & professional	4.49 (4.45, 4.53)		4.47 (4.43, 4.51)		4.52 (4.48, 4.57)		4.50 (4.47, 4.52)			
Intermediate	4.53 (4.49, 4.57)		4.51 (4.46, 4.55)		4.57 (4.53, 4.61)		4.53 (4.51, 4.55)			
Routine & manual	4.52 (4.48, 4.56)		4.47 (4.43, 4.51)		4.56 (4.52, 4.59)		4.51 (4.49, 4.54)			
Economically inactive	4.51 (4.46, 4.57)		4.56 (4.50, 4.61)		4.54 (4.50, 4.59)		4.53 (4.51, 4.56)			
Unclassified	4.48 (4.36, 4.60)		4.51 (4.44, 4.57)		4.49 (4.40, 4.59)		4.52 (4.48, 4.57)			
% difference per NSSEC†	0.17 (−0.27, 0.60)	0.45	0.40 (−0.01, 0.82)	0.05	0.09 (−0.33, 0.51)	0.68	0.19 (−0.02, 0.41)	0.08	0.51	
% difference per NS-SEC€	0.36 (−0.25, 0.97)	0.24	−0.01 (−0.60, 0.58)	0.98	0.32 (−0.27, 0.91)	0.28	0.16 (−0.14, 0.47)	0.29		
p-value NS-SEC (nominal)*		0.73		0.02		0.42		0.06		0.32
**Insulin resistance (HOMA-IR)**										
Managerial & professional	0.72 (0.68, 0.77)		0.98 (0.91, 1.05)		0.99 (0.92, 1.08)		0.90 (0.86, 0.94)			
Intermediate	0.79 (0.74, 0.85)		0.99 (0.92, 1.07)		1.02 (0.95, 1.10)		0.93 (0.89, 0.97)			
Routine & manual	0.79 (0.73, 0.85)		0.97 (0.90, 1.05)		1.03 (0.96, 1.10)		0.92 (0.89, 0.96)			
Economically inactive	0.87 (0.79, 0.96)		0.88 (0.80, 0.96)		1.00 (0.92, 1.08)		0.91 (0.87, 0.95)			
Unclassified	0.66 (0.53, 0.83)		0.97 (0.86, 1.10)		1.10 (0.93, 1.31)		0.93 (0.86, 1.01)			
% difference per NSSEC†	5.69 (2.01, 9.51)	0.002	−2.75 (−5.99, 0.61)	0.10	0.29 (−3.08, 3.79)	0.86	0.52 (−1.25, 2.32)	0.56	0.002	
% difference per NS-SEC€	4.92 (−0.16, 10.26)	0.05	−0.29 (−5.00, 4.65)	0.90	1.86 (−2.92, 6.87)	0.44	1.41 (−1.09, 3.97)	0.26		
p-value NS-SEC (nominal)*		0.01		0.17		0.85		0.63		0.02

Mean: means adjusted for sex, age, observer, month and school (random effect). Missing values: glucose (n = 33), insulin resistance (n = 151).

95% CI: 95% confidence interval of the mean.

¶ Estimates adjusted for ethnicity (all groups included).

‡ interaction test of NS-SEC and main ethnic groups (white European, black African-Caribbean, South Asian) and excluding “unclassified” NS-SEC group fitting NS-SEC as an ordinal variable.

§ interaction test of NS-SEC and main ethnic groups (white European, black African-Caribbean, South Asian) and excluding “unclassified” NS-SEC group fitting NS-SEC as a categorical variable.

† per NS-SEC decrement from professional to economically inactive (excluding unclassified group).

€ per NS-SEC decrement from professional to routine &manual (excluding economically inactive and unclassified groups).

* p-value for NS-SEC fitted as an unordered nominal variable (excluding unclassified group).

Percentage variance due to school differences: HbA1c 5.8%, glucose 7.5%, insulin resistance 6.7%.

**Table 5:** Adjusted means and mean differences in triglyceride, HDL-cholesterol and C-reactive protein by NS-SEC and ethnic group.

Outcome & NS-SEC	White European (n = 1158)		Black African-Caribbean (n = 1201)		South Asian (n = 1314)		All CHASE (n = 4804)¶		Difference between WE, AC & SA groups	
	Mean (95% CI)	*P*	Mean (95% CI)	*P*	Mean (95% CI)	*P*	Mean (95% CI)	*P*	*P* ‡	*P* §
**Triglyceride (mmol/L)**										
Managerial & professional	0.75 (0.72, 0.78)		0.71 (0.68, 0.74)		0.88 (0.84, 0.93)		0.79 (0.77, 0.81)			
Intermediate	0.80 (0.77, 0.84)		0.72 (0.69, 0.76)		0.93 (0.89, 0.98)		0.82 (0.80, 0.84)			
Routine & manual	0.82 (0.79, 0.86)		0.71 (0.68, 0.74)		0.87 (0.83, 0.90)		0.80 (0.78, 0.82)			
Economically inactive	0.81 (0.77, 0.86)		0.71 (0.67, 0.75)		0.91 (0.86, 0.95)		0.82 (0.79, 0.84)			
Unclassified	0.79 (0.69, 0.91)		0.69 (0.64, 0.74)		0.98 (0.89, 1.09)		0.82 (0.78, 0.86)			
% difference per NSSEC†	3.17 (0.96, 5.42)	0.004	−0.35 (−2.39, 1.73)	0.73	−0.22 (−2.28, 1.89)	0.84	0.82 (−0.26, 1.91)	0.13	0.03	
% difference per NS-SEC€	5.14 (2.01, 8.37)	0.001	0.01 (−2.89, 3.00)	0.99	−1.33 (−4.19, 1.62)	0.36	0.91 (−0.613, 2.46)	0.23		
p-value NS-SEC (nominal)*		0.02		0.91		0.04		0.10		0.07
**HDL-cholesterol (mmol/L)**										
Managerial & professional	1.51 (1.48, 1.54)		1.51 (1.48, 1.54)		1.46 (1.43, 1.50)		1.50 (1.48, 1.52)			
Intermediate	1.48 (1.45, 1.52)		1.53 (1.49, 1.57)		1.44 (1.40, 1.47)		1.48 (1.46, 1.50)			
Routine & manual	1.49 (1.46, 1.53)		1.49 (1.46, 1.53)		1.45 (1.42, 1.48)		1.48 (1.46, 1.50)			
Economically inactive	1.45 (1.41, 1.50)		1.52 (1.48, 1.56)		1.43 (1.40, 1.47)		1.46 (1.44, 1.48)			
Unclassified	1.52 (1.41, 1.63)		1.53 (1.47, 1.59)		1.43 (1.35, 1.51)		1.48 (1.44, 1.51)			
% difference per NSSEC†	−1.03 (−2.17, 0.11)	0.07	−0.13 (−1.23, 0.98)	0.81	−0.50 (−1.59, 0.61)	0.37	−0.87 (−1.43, −0.30)	0.002	0.51	
% difference per NS-SEC€	−0.66 (−2.26, 0.96)	0.41	−0.53 (−2.09, 1.06)	0.50	−0.37 (−1.93, 1.21)	0.63	−0.78 (−1.58, 0.03)	0.05		
p-value NS-SEC (nominal)*		0.28		0.58		0.58		0.02		0.47
**C-reactive protein (mg/L)**										
Managerial & professional	0.39 (0.34, 0.45)		0.51 (0.45, 0.59)		0.56 (0.47, 0.66)		0.48 (0.45, 0.52)			
Intermediate	0.39 (0.34, 0.45)		0.55 (0.46, 0.64)		0.66 (0.57, 0.76)		0.52 (0.48, 0.57)			
Routine & manual	0.42 (0.36, 0.50)		0.59 (0.50, 0.69)		0.62 (0.54, 0.70)		0.53 (0.49, 0.57)			
Economically inactive	0.46 (0.38, 0.56)		0.42 (0.35, 0.51)		0.55 (0.46, 0.65)		0.49 (0.44, 0.54)			
Unclassified	0.47 (0.30, 0.76)		0.46 (0.36, 0.60)		0.55 (0.38, 0.79)		0.47 (0.39, 0.55)			
% difference per NSSEC†	5.68 (−2.05, 14.02)	0.15	−2.61 (−9.47, 4.78)	0.47	−0.97 (−7.90, 6.47)	0.79	1.13 (−2.59, 5.00)	0.55	0.29	
% difference per NS-SEC€	4.37 (−6.07, 15.95)	0.42	7.20 (−3.32, 18.86)	0.18	4.58 (−5.56, 15.81)	0.38	4.79 (−0.60, 10.47)	0.08		
p-value NS-SEC (nominal)*		0.60		0.06		0.29		0.25		0.23

Mean: means adjusted for sex, age, observer, month and school (random effect). Missing values: CRP (n = 159).

95% CI: 95% confidence interval of the mean.

¶ Estimates adjusted for ethnicity (all groups included).

‡ interaction test of NS-SEC and main ethnic groups (white European, black African-Caribbean, South Asian) and excluding “unclassified” NS-SEC group fitting NS-SEC as an ordinal variable.

§ interaction test of NS-SEC and main ethnic groups (white European, black African-Caribbean, South Asian) and excluding “unclassified” NS-SEC group fitting NS-SEC as a categorical variable.

† per NS-SEC decrement from professional to economically inactive (excluding unclassified group).

€ per NS-SEC decrement from professional to routine &manual (excluding economically inactive and unclassified groups).

* p-value for NS-SEC fitted as an unordered nominal variable (excluding unclassified group).

Percentage variance due to school differences: Triglyceride 5.3%, HDL-cholesterol 1.8%, C-reactive protein 1.6%.

#### Analyses treating NS-SEC3 as an ordinal variable

In ordinal analyses including children from all ethnic groups (and adjusting for differences between ethnic groups) children were 0.17% (95% CI 0.04, 0.30%) shorter (Table 2) and HDL-cholesterol levels were 0.87% (95%CI 0.30, 1.43%) lower (Table 5) with each NS-SEC3 decrement. Although the associations between NS-SEC3, height and HDL-cholesterol, did not differ significantly between ethnic groups, there was evidence of marked heterogeneity between ethnic groups in the ordinal relations between NS-SEC3 and several other T2DM risk factors, including weight, ponderal index, sum of skinfolds, fat mass index, waist circumference, insulin resistance and triglyceride (Tables 2–5). Overall, associations between NS-SEC3 and T2DM risk factors were most marked among white Europeans, among whom lower NS-SEC3 was significantly related to higher adiposity levels (ponderal index, sum of skinfolds, fat mass index, waist circumference), higher insulin resistance and higher triglyceride levels. Among black African-Caribbean children, lower NS-SEC3 status was statistically significantly associated with lower height, weight and ponderal index and weakly associated with lower levels of insulin resistance and triglyceride (i.e. opposite in direction to those in white Europeans). There was no evidence of any trends in South Asian children.

These results were not materially affected by using the alternative analytic approach of combining the economically inactive group with the lowest employed group in the NS-SEC3 categories (routine and manual) (data not presented). Exclusion of the economically inactive group from analyses had little effect on the associations between NS-SEC3 and T2DM risk factors in white Europeans (Tables 2–5). However, the previously observed associations between low NS-SEC3 and lower height, weight, ponderal index, insulin resistance and triglyceride among black African-Caribbeans were less apparent and the interactions observed between NS-SEC3 and ethnicity were no longer statistically significant, with the exception of that for triglyceride (p = 0.01).

#### Analyses treating NS-SEC3 and NS-SEC5 as nominal variables

Analyses that showed linear trends with NS-SEC3 generally also showed heterogeneity by NS-SEC3 as a nominal variable. There was evidence of statistically significant variation for the same T2DM risk factors in which trends were observed in ordinal analyses (Tables 2 to [Table pone-0032619-t003] [Table pone-0032619-t004] 5). In particular, for the whole study population there was evidence of variation in height and HDL-cholesterol between NS-SEC3 groups; variation in ponderal index and fat mass index was also apparent. There was also marked heterogeneity between ethnic groups in the associations between NS-SEC3 and adiposity (especially for weight, ponderal index, fat mass index and insulin resistance (Tables 2–4), consistent with the results of ordinal analyses. Among white Europeans, there was strong evidence of variation in adiposity (ponderal index, sum of skinfolds, fat mass index, waist circumference), insulin resistance and triglyceride among NS-SEC3 groups. Black African-Caribbean children showed evidence of associations between NS-SEC and adiposity, with lower adiposity levels among the economically inactive group, particularly for ponderal index and fat mass index. South Asian children showed no consistent associations between NS-SEC and T2DM risk factors.

Analyses using NS-SEC5 treated as a nominal variable (Tables S1 and S2) showed similar risk factor patterns to NS-SEC3, with evidence of differences in associations between ethnic groups for associations with adiposity (particularly ponderal index), insulin resistance and triglyceride (at marginal levels of statistical significance). Again there was marked variation in adiposity, insulin resistance and triglyceride between NS-SEC5 groups within the white European group; lower NS-SEC was related to higher levels of adiposity (ponderal index, sum of skinfolds, fat mass index, waist circumference), insulin resistance and triglyceride. In addition, white European children whose parents were defined as ‘small employer/own account’ had greater adiposity, insulin resistance, and triglyceride levels than children from managerial/professional families. In contrast, NS-SEC5 showed little consistent association with T2DM risk factors in either the black African-Caribbean or South Asian groups.

These results were again unaffected by combining the economically inactive group with the lowest employed group in the NS-SEC3 or NS-SEC5 categories (data not presented). When the economically inactive group was excluded from analyses, strong evidence of variation in adiposity markers and triglyceride between NS-SEC3 and NS-SEC5 categories were still apparent in the white Europeans, though there was little evidence of similar variation in black African-Caribbeans or South Asians. Evidence of heterogeneity in associations between NS-SEC categories in different ethnic groups was still apparent for triglycerides but not for adiposity or insulin resistance.

### NS-SEC and type 2 diabetes risk factors in ethnic sub-groups

The associations between NS-SEC3 and T2DM risk factors were examined separately in black African and black Caribbean children, and separately in Indian, Pakistani and Bangladeshi children (Tables S3 and S4). There was little evidence that the (generally weak) associations between NS-SEC3 and T2DM risk factors in these ethnic sub-groups differed between black Africans and black Caribbeans, or between Indians, Pakistanis or Bangladeshis, either when NS-SEC3 was treated as an ordinal or nominal variable. Among the associations referred to above, there was some evidence that the associations between higher NS-SEC3 and greater adiposity (particularly ponderal index and fat mass index) and insulin resistance were stronger among black Africans than black Caribbeans. However, there was no strong evidence that any associations between NS-SEC and T2DM risk factors differed between Indian, Pakistani or Bangladeshi children (Tables S3 and S4).

### Ethnic differences in type 2 diabetes risk factors: influence of NS-SEC

As previously reported [15], compared to white European children, South Asian children had higher sum of skinfolds, fat mass index, HbA1c, glucose, insulin resistance, triglycerides and C-reactive protein and lower HDL-cholesterol (Table S5). Black African-Caribbean children had less marked increases in HbA1c, insulin resistance and C-reactive protein, but conversely, had lower triglycerides and higher HDL-cholesterol; adiposity levels were not consistently increased (Table S5).

Adjustment for NS-SEC5 made no material difference to the size and direction of these ethnic differences (Table S5). Further analyses including parental education and household amenities had no further effect on these differences (data not presented). The stratified analysis showed that the higher levels of skinfolds, fat mass index, insulin resistance and triglyceride levels observed in South Asians compared with white Europeans were particularly marked in the higher NS-SEC3 groups (Table S6). Similarly, the higher fat mass index and insulin resistance observed in black African-Caribbeans compared with white Europeans were particularly marked in the higher NS-SEC3 groups; in contrast, the lower triglyceride levels were particularly marked in the lower NS-SEC3 groups.

## Discussion

### Main findings

In this school-based study of 9–10 year old children living in London, Birmingham and Leicester, SEP (measured by NS-SEC) was associated with adiposity and insulin resistance, although these associations appeared to vary between different ethnic groups. White European children from lower NS-SEC groups had higher levels of adiposity and insulin resistance, while black African-Caribbean children from lower NS-SEC groups (particularly the economically inactive group) had lower adiposity levels, findings that were particularly marked in African children. Little association was apparent for South Asian children and there was little evidence of heterogeneity between South Asian groups. Marked ethnic differences in T2DM risk factors were unaffected by adjustment for NS-SEC5; in stratified analyses, ethnic differences in fat mass index and insulin levels in particular were largest in the higher socio-economic groups.

### Comparison with other studies

In adults, low SEP is consistently related to higher T2DM prevalence and associated morbidity and mortality, particularly in white Europeans in high income countries including the UK [6], [7]. However, data on socio-economic gradients in T2DM in UK South Asians and black African-Caribbeans are limited; current evidence to date suggests that the inverse association between SEP and T2DM and its risk factors are weaker in South Asians than white Europeans [2], [3], [8]. Previous studies of socio-economic gradients in T2DM in black African-Caribbeans have been less consistent and depend on the SEP measure used. Using the Registrar General's occupational social class, the National Survey of Ethnic Minorities failed to show a clear association with diabetes prevalence in Caribbean adults, but found that tenants were more likely to report diabetes than homeowners [3]. The Health Survey for England reported an inverse association between income and T2DM among Caribbean adults [2].

In UK children, there is little direct evidence on associations between SEP and T2DM risk factors. In the present study, most variation in T2DM risk factors (particularly adiposity) occurred at individual rather than school level, a finding consistent with observations in previous school-based trials of obesity prevention in children [23]. Associations between low SEP and greater adiposity have been increasingly apparent in recent studies, particularly in white Europeans [18] and have recently been reported in 10 year-olds in the large ALSPAC Study (a predominantly white European population) based on DXA total body fat measurements [24]. This pattern, though not consistently observed in studies before 2000 [17], [18], is consistent with our own findings and suggests that associations between low SEP and greater adiposity are present in the first decade of life, particularly in UK white European children; similar patterns have also been reported in studies of predominantly white children in the US [25]–[27]. No UK studies have to our knowledge reported patterns of associations between SEP and insulin resistance in children, but our observations of an inverse association in white Europeans are consistent with the results of the European Youth Heart Study in Denmark [28], studies in the USA [29], and with the socio-economic gradient in T2DM seen for UK adults [5], [8]. Earlier UK reports have shown little consistent evidence of associations between SEP and blood lipids in white Europeans [17]; our finding of an association between low SEP and high triglyceride is however consistent with the increasing evidence of associations between low SEP and greater adiposity in other studies [18]. The lack of an association between SEP and T2DM risk factors in South Asian children is consistent with adult data [2]–[4]. The associations between low SEP and lower adiposity and insulin resistance among black African-Caribbean children, though less consistent with adult studies, are consistent with the patterns observed for adiposity and insulin resistance patterns in children in less affluent countries undergoing socio-economic transition [28], [30] and with adiposity patterns in African American children in the US [25].

### Strengths and limitations

Particular strengths of CHASE were its large size (designed to detect modest differences in T2DM risk factors between main ethnic groups), strong representation of ethnic minority groups and detailed measurements, particularly including adiposity indices that are more suitable than body mass index for studies of ethnically diverse children [31]–[33]. Insulin resistance was estimated from fasting glucose and insulin concentrations using the homeostatic model assessment (HOMA) method, a validated measure of insulin resistance shown to be associated with an increased risk of developing T2DM in longitudinal studies and appropriate for use in large, epidemiological studies where only a single fasting plasma sample is required [34]. Results using the HOMA-IR method, which has also been validated in children [35], were consistent with findings using fasting insulin (data not presented).

The study sampled schools from three cities in which most UK South Asians and black African-Caribbeans reside [36] and included similar numbers of South Asian children of Indian, Pakistani and Bangladeshi origin and of black African-Caribbean children of black African and black Caribbean origin. Thus, the study is likely to have stronger general representation of ethnic minority groups than white Europeans, higher proportions of whom live in other parts of the UK. The effect of non-participation at school level (30% of invited schools, with replacement of each non-participant school with a school of similar characteristics) is difficult to assess; decisions were often made for reasons which appeared to be unrelated to pupil characteristics (e.g. impending school inspections or senior staff changes). The restriction of the study to state schools and the moderate individual response rates may have limited representation of high SEP and low SEP participants respectively. However, response rates were very similar in most ethnic groups and comparisons of the characteristics of responders and non-responders provided little evidence of selection bias. The proportions of white European children in CHASE whose highest NS-SEC parent was in managerial/professional (31%) and routine/manual (24%) occupations were reassuringly similar to the 2001 Census (27% and 28% respectively), though intermediate occupations were somewhat overrepresented (28% vs 17% in the Census) [37]. In black African-Caribbeans, professional/managerial occupations were over-represented (33% vs 25% in Census), while among South Asians, routine/manual occupations were over-represented (35% vs 24% in Census). Nevertheless, the proportion of children living in a “workless household” (17%) was comparable to the overall Labour Force Survey (LFS) estimate of 16%, while the prevalences in specific ethnic groups closely matched national data for all groups except black Caribbeans [38]. Although the white European population in CHASE may not be fully representative of the wider UK white European population, the associations between low NS-SEC and higher T2DM risk markers observed in white European children in CHASE (particularly affecting adiposity, insulin and triglyceride) were reassuringly consistent with those in previous recent reports [18], [23].

Our main analyses focused on the three main ethnic groups living in the UK – white Europeans, South Asians and black African-Caribbeans. These groups were used because of their common origins and similar within-group T2DM risks (very high among South Asian groups, moderately high among black Africans and black Caribbeans). However, supplementary analyses examining differences in the relations between SEP and T2DM risk factors among Indian, Pakistani and Bangladeshi children and among black Africans and black Caribbeans were also carried out. We did not find strong evidence for heterogeneity in the associations between NS-SEC and T2DM risk factors amongst South Asian children, although some heterogeneity was apparent for black African-Caribbean children, particularly for adiposity. However, even with a study population of this size, statistical power for the detection of differences is limited.

The use of the NS-SEC (a recently developed national occupational classification taking account of current employment patterns and including non-employed categories) for defining SEP is a further strength of the study. The NS-SEC places people into classes according to their occupational title and employment relations and conditions such as employers or employees, whether they have a wage or salary, levels of autonomy and prospects for promotion [39]. As such, the NS-SEC is considered a better theoretical indicator of SEP than the Registrar General's Social Class (based primarily on occupation) and also may be better suited to studying health inequalities in ethnic groups [39]. The NS-SEC3 can be treated as an ordinal or nominal variable, while the NS-SEC5 is used primarily as a nominal variable. The validity of the NS-SEC in different ethnic groups in the present study is supported by the association between low NS-SEC and shorter stature (a key marker of childhood nutrition) in the whole study population, with no strong evidence of heterogeneity in this association between ethnic groups (Table 2). Its identification of small employers and own account workers (self-employed) may be particularly relevant for South Asians [39]. In accordance with official NS-SEC guidance [22], the substantial group of economically inactive subjects were classified into a separate low SEP group placed below the lowest employed group; similar results were obtained using the alternative recommended strategy of merging the economically inactive group with the lowest employed group [22]. This approach ensured the widest possible representation of the range of SEP in this study population and maximized the number of participants included in analysis.

### Implications

An important finding of the present study is that while among white Europeans, low NS-SEC groups have greater adiposity and insulin resistance, the patterns of association between SEP and T2DM risk factors in other ethnic groups within the UK population may differ. These patterns were apparent whether NS-SEC was treated as an ordinal or nominal variable, and whether fitted as a three class or five class variable. They were also similar when the outcome variables were treated as dichotomous (focussing on the top fifths of the distributions of adiposity, insulin and blood lipids). However, the results (particularly the evidence that the associations between NS-SEC and T2DM risk factors differed between ethnic groups) were dependent on the inclusion of the economically inactive groups in the analysis; exclusion of this group markedly reduced the evidence of heterogeneity in associations between NS-SEC and T2DM risk factors between ethnic groups. This could simply reflect the reduced number of groups and study participants and the more limited range of social circumstances and the consequent reduction in statistical power, or it could reflect the exclusion of particular economically inactive groups, perhaps especially the black African-Caribbean group, with low levels of adiposity and insulin resistance. However, the observed patterns are consistent with earlier reports of the differing associations between SEP and adiposity in black and white populations in the USA [25] and with evidence from other sources which suggest that associations between SEP and chronic disease risks differ between times, places and population groups [40].

The associations between low SEP and higher levels of T2DM risk factors in white European children are consistent with the socio-economic patterning of childhood obesity reported in other studies both in the UK [24] and more widely [18], [30] and with the socio-economic patterning of chronic disease (particularly T2DM and cardiovascular disease) observed in current and previous adult generations, reflecting the later stage of the socioeconomic transition [41]. The weaker associations observed in South Asian and black African-Caribbean children, (with some evidence of an opposite association among black African-Caribbean children) would be consistent with earlier stages of the socio-economic transition where adverse exposures are less concentrated among lower SEP groups than in later stages [41]–[44] a pattern which has also been reported for adiposity and insulin resistance in children in less affluent countries [28], [30] and for adiposity in African American children [25].

The findings in our study could reflect the evolution of socially determined exposures such as dietary characteristics (particularly total calorie and fat intakes) and physical inactivity (influenced by patterns of family car use). However, overall associations between SEP and physical activity are weak and patterns in individual ethnic groups do not correspond to those described here for adiposity and insulin resistance (C Thomas, unpublished data). Dietary factors could therefore be important and need further exploration. As diets have changed in migrant populations to reflect those of the host population, particularly among younger age groups [45], [46] investigation into dietary patterns and practices, including food purchasing and cooking patterns and the extent of dietary acculturation (perhaps especially in black African-Caribbean children from low NS-SEC families) could shed further light on the role of health behaviours in explaining ethnic differences in T2DM risk factors.

The analyses examining the impact of NS-SEC adjustment on ethnic differences in T2DM risk factors suggested that ethnic differences in SEP did not directly account for ethnic differences in T2DM risk factors. Despite its strengths, the NS-SEC classification cannot represent all dimensions of SEP and it is therefore possible that these analyses are affected by residual confounding [3]. However, this possibility is made less likely by the very limited effect of NS-SEC adjustment on the size of ethnic differences, by the minimal effect of additional adjustments for parental education and household amenity score, as well as by the evidence that the association between NS-SEC and T2DM risk factors differs by ethnic groups, which render simple approaches to NS-SEC adjustment potentially inappropriate. This conclusion was further strengthened by the results of stratified analyses, which showed that ethnic differences were apparent at all SEP levels, although they were particularly marked in the highest SEP groups, which argues against the “underclass” hypothesis [47].

These results have important implications for strategies for early prevention of T2DM risk. Ethnic differences in NS-SEC do not appear to explain why UK black African-Caribbean and South Asian children are more adipose and insulin resistant than white European children. Moreover, ethnic differences in T2DM risk markers appear to be largest in the most affluent socio-economic groups. These findings highlight important implications for strategies in early T2DM prevention. First, they suggest that strategies aimed at reducing socio-economic inequalities in emerging T2DM risk in childhood will not be effective in reducing ethnic differences in T2DM risk, a conclusion also reached by other investigators [25]. However, efforts to reduce social inequalities in T2DM risk could be particularly important in white European children, in whom low SEP is strongly associated with T2DM risk factors. Efforts to identify and control the determinants of adiposity and insulin resistance among white European children from low SEP groups are a key priority for early T2DM prevention.

## Supporting Information

Table S1 Adjusted mean physical measures by NS-SEC (5-class) and main ethnic group.
(DOCX)

Table S2 Adjusted mean blood based measures by NS-SEC (5 class) and main ethnic group.
(DOCX)

Table S3 Adjusted mean physical measures by NS-SEC and ethnic sub-group.
(DOCX)

Table S4 Adjusted mean blood based measures by NS-SEC and ethnic sub-group.
(DOCX)

Table S5 Ethnic differences in type 2 diabetes risk factors compared to white European children with and without adjustment for NS-SEC.
(DOCX)

Table S6 Ethnic differences (compared to white European) in type 2 diabetes risk factors by NS-SEC group.
(DOCX)
