# Supplementary material for: Socio-Economic Position and Type 2 Diabetes Risk Factors: Patterns in UK Children of South Asian, Black African-Caribbean and White European Origin
Source: PLoS One. 2012 Mar 7;7(3):e32619. doi: 10.1371/journal.pone.0032619 (PMC3296720; doi:10.1371/journal.pone.0032619)
Supplement: Table S1 — Adjusted mean physical measures by NS-SEC (5-class) and main ethnic group. (DOCX) [file pone.0032619.s001.docx]

**Table S1. Adjusted mean physical measures by NS-SEC (5-class) and main ethnic group**

| **Outcome & NS-SEC** | **White European (n=1158)** | | **Black African-Caribbean (n=1201)** | | **South Asian (n=1314)** | | **All CHASE (n=4804)** ¶ | | **Difference between WE, AC & SA groups** |
| --- | --- | --- | --- | --- | --- | --- | --- | --- | --- |
|  | **mean (95% CI)** | ***P*-value** | **mean (95% CI)** | ***P*-value** | **mean (95% CI)** | ***P*-value** | **mean (95% CI)** | ***P*-value** | ***P*-value§** |
| **Height (cm)** |  |  |  |  |  |  |  |  |  |
| Managerial & professional | 139.1 (138.5, 139.8) |  | 143.3 (142.6, 144.0) |  | 138.5 (137.7, 139.3) |  | 140.3 (139.9, 140.6) |  |  |
| Intermediate | 139.0 (138.0, 140.0) |  | 142.8 (141.7, 143.8) |  | 138.8 (137.8, 139.9) |  | 140.1 (139.6, 140.6) |  |  |
| Small employers & own account | 139.8 (138.8, 140.8) |  | 142.6 (141.3, 144.0) |  | 138.4 (137.4, 139.4) |  | 139.8 (139.3, 140.4) |  |  |
| Lower supervisory & technical | 139.1 (137.5, 140.7) |  | 140.5 (138.5, 142.5) |  | 139.0 (137.0, 141.0) |  | 139.7 (138.7, 140.6) |  |  |
| Semi-routine & routine | 138.3 (137.4, 139.1) |  | 143.3 (142.5, 144.1) |  | 138.6 (138.0, 139.3) |  | 139.8 (139.4, 140.2) |  |  |
| Economically inactive | 139.0 (138.0, 140.0) |  | 141.5 (140.6, 142.5) |  | 138.2 (137.4, 139.0) |  | 139.5 (139.1, 140.0) |  |  |
| Unclassified | 138.7 (136.4, 141.0) |  | 144.3 (142.9, 145.6) |  | 139.0 (137.2, 140.8) |  | 140.5 (139.7, 141.3) |  |  |
| p-value NS-SEC (nominal)* |  | 0.34 |  | 0.01 |  | 0.95 |  | 0.17 | 0.09 |
| **Weight (kg)** |  |  |  |  |  |  |  |  |  |
| Managerial & professional | 34.7 (33.9, 35.5) |  | 39.3 (38.4, 40.2) |  | 33.9 (33.0, 34.9) |  | 36.0 (35.6, 36.5) |  |  |
| Intermediate | 34.2 (32.9, 35.4) |  | 39.3 (37.9, 40.7) |  | 34.1 (32.9, 35.4) |  | 36.0 (35.4, 36.7) |  |  |
| Small employers & own account | 35.8 (34.6, 37.1) |  | 38.9 (37.1, 40.7) |  | 34.7 (33.5, 35.9) |  | 36.0 (35.3, 36.7) |  |  |
| Lower supervisory & technical | 35.3 (33.4, 37.3) |  | 38.5 (35.9, 41.3) |  | 35.1 (32.7, 37.7) |  | 36.2 (35.0, 37.4) |  |  |
| Semi-routine & routine | 35.8 (34.7, 37.0) |  | 39.2 (38.1, 40.3) |  | 34.7 (33.9, 35.6) |  | 36.3 (35.8, 36.8) |  |  |
| Economically inactive | 36.0 (34.7, 37.3) |  | 36.1 (34.9, 37.3) |  | 33.8 (32.9, 34.8) |  | 35.5 (34.9, 36.1) |  |  |
| Unclassified | 34.4 (31.7, 37.3) |  | 39.3 (37.6, 41.1) |  | 34.8 (32.6, 37.0) |  | 36.1 (35.0, 37.1) |  |  |
| p-value NS-SEC (nominal)* |  | 0.16 |  | 0.001 |  | 0.64 |  | 0.41 | 0.02 |
| **Ponderal index (kg/m^3^)** |  |  |  |  |  |  |  |  |  |
| Managerial & professional | 12.9 (12.7, 13.1) |  | 13.3 (13.1, 13.6) |  | 12.8 (12.5, 13.0) |  | 13.1 (12.9, 13.2) |  |  |
| Intermediate | 12.7 (12.4, 13.1) |  | 13.5 (13.1, 13.8) |  | 12.8 (12.4, 13.1) |  | 13.1 (12.9, 13.3) |  |  |
| Small employers & own account | 13.1 (12.8, 13.4) |  | 13.4 (12.9, 13.8) |  | 13.1 (12.8, 13.4) |  | 13.2 (13.0, 13.3) |  |  |
| Lower supervisory & technical | 13.1 (12.6, 13.7) |  | 13.9 (13.2, 14.6) |  | 13.1 (12.4, 13.8) |  | 13.3 (13.0, 13.6) |  |  |
| Semi-routine & routine | 13.6 (13.3, 13.9) |  | 13.3 (13.0, 13.6) |  | 13.0 (12.8, 13.3) |  | 13.3 (13.2, 13.4) |  |  |
| Economically inactive | 13.4 (13.1, 13.8) |  | 12.7 (12.4, 13.0) |  | 12.8 (12.5, 13.1) |  | 13.1 (12.9, 13.2) |  |  |
| Unclassified | 12.9 (12.2, 13.7) |  | 13.1 (12.7, 13.5) |  | 12.9 (12.4, 13.5) |  | 13.0 (12.7, 13.3) |  |  |
| p-value NS-SEC (nominal)* |  | 0.001 |  | 0.004 |  | 0.41 |  | 0.10 | 0.001 |
| **Sum of skinfolds (mm)** |  |  |  |  |  |  |  |  |  |
| Managerial & professional | 38.4 (36.5, 40.4) |  | 39.4 (37.5, 41.5) |  | 41.9 (39.5, 44.4) |  | 40.4 (39.3, 41.6) |  |  |
| Intermediate | 37.1 (34.3, 40.0) |  | 40.4 (37.4, 43.5) |  | 42.4 (39.2, 45.7) |  | 40.8 (39.2, 42.4) |  |  |
| Small employers & own account | 40.7 (37.8, 43.7) |  | 39.8 (36.1, 43.9) |  | 45.3 (42.1, 48.7) |  | 41.2 (39.6, 42.9) |  |  |
| Lower supervisory & technical | 40.3 (35.8, 45.3) |  | 41.5 (35.9, 48.0) |  | 45.4 (39.1, 52.7) |  | 41.5 (38.7, 44.5) |  |  |
| Semi-routine & routine | 43.2 (40.5, 46.1) |  | 41.1 (38.7, 43.6) |  | 43.3 (41.2, 45.5) |  | 42.1 (40.8, 43.3) |  |  |
| Economically inactive | 43.5 (40.4, 46.8) |  | 38.7 (36.1, 41.5) |  | 40.6 (38.2, 43.1) |  | 40.6 (39.2, 42.0) |  |  |
| Unclassified | 36.5 (30.8, 43.2) |  | 39.5 (35.9, 43.4) |  | 42.6 (37.4, 48.5) |  | 39.9 (37.5, 42.4) |  |  |
| p-value NS-SEC (nominal)* |  | 0.005 |  | 0.80 |  | 0.25 |  | 0.44 | 0.12 |
| **Fat mass index (kg/m^5^)** |  |  |  |  |  |  |  |  |  |
| Managerial & professional | 1.64 (1.55, 1.73) |  | 1.81 (1.72, 1.92) |  | 1.79 (1.68, 1.91) |  | 1.75 (1.70, 1.81) |  |  |
| Intermediate | 1.54 (1.42, 1.67) |  | 1.87 (1.73, 2.03) |  | 1.80 (1.66, 1.96) |  | 1.78 (1.70, 1.86) |  |  |
| Small employers & own account | 1.72 (1.59, 1.86) |  | 1.88 (1.69, 2.09) |  | 1.94 (1.80, 2.10) |  | 1.85 (1.77, 1.93) |  |  |
| Lower supervisory & technical | 1.75 (1.54, 1.99) |  | 2.04 (1.75, 2.39) |  | 2.01 (1.71, 2.37) |  | 1.89 (1.75, 2.04) |  |  |
| Semi-routine & routine | 1.86 (1.74, 2.00) |  | 1.84 (1.72, 1.96) |  | 1.90 (1.80, 2.00) |  | 1.85 (1.79, 1.91) |  |  |
| Economically inactive | 1.79 (1.66, 1.94) |  | 1.66 (1.54, 1.78) |  | 1.78 (1.67, 1.90) |  | 1.77 (1.70, 1.84) |  |  |
| Unclassified | 1.64 (1.37, 1.96) |  | 1.77 (1.60, 1.96) |  | 1.78 (1.55, 2.05) |  | 1.76 (1.65, 1.88) |  |  |
| p-value NS-SEC (nominal)* |  | 0.01 |  | 0.09 |  | 0.27 |  | 0.05 | 0.06 |
| **Waist circumference (cm)** |  |  |  |  |  |  |  |  |  |
| Managerial & professional | 63.1 (62.2, 64.0) |  | 64.4 (63.4, 65.3) |  | 62.5 (61.5, 63.6) |  | 63.5 (63.0, 64.0) |  |  |
| Intermediate | 62.5 (61.1, 63.9) |  | 64.4 (63.0, 65.8) |  | 62.7 (61.3, 64.1) |  | 63.5 (62.8, 64.2) |  |  |
| Small employers & own account | 64.8 (63.4, 66.2) |  | 64.3 (62.5, 66.2) |  | 63.6 (62.3, 65.0) |  | 64.1 (63.3, 64.8) |  |  |
| Lower supervisory & technical | 64.7 (62.5, 66.9) |  | 65.3 (62.6, 68.1) |  | 64.3 (61.6, 67.2) |  | 64.6 (63.3, 65.9) |  |  |
| Semi-routine & routine | 65.0 (63.8, 66.2) |  | 64.5 (63.4, 65.6) |  | 63.7 (62.8, 64.6) |  | 64.3 (63.8, 64.9) |  |  |
| Economically inactive | 65.2 (63.8, 66.6) |  | 62.7 (61.5, 64.0) |  | 62.4 (61.3, 63.5) |  | 63.6 (63.0, 64.3) |  |  |
| Unclassified | 63.3 (60.2, 66.5) |  | 64.1 (62.4, 65.9) |  | 63.0 (60.7, 65.5) |  | 63.5 (62.4, 64.7) |  |  |
| p-value NS-SEC (nominal)* |  | 0.01 |  | 0.30 |  | 0.26 |  | 0.17 | 0.12 |

Mean: adjusted for sex, age, observer, month and school (random effect). Missing values: skinfolds (n=12), fat mass index (n=64), waist circumference (n=1).

95% CI: 95% confidence interval for the mean

¶ Estimates obtained from models containing all ethnic groups

§interaction test of NS-SEC and main ethnic groups (white European, black African-Caribbean, South Asian) and excluding "unclassified" NS-SEC group fitting NS-SEC as a categorical variable

*p-value for NS-SEC fitted as an unordered nominal variable (excluding unclassified group)
