# Supplementary material for: Socio-Economic Position and Type 2 Diabetes Risk Factors: Patterns in UK Children of South Asian, Black African-Caribbean and White European Origin
Source: PLoS One. 2012 Mar 7;7(3):e32619. doi: 10.1371/journal.pone.0032619 (PMC3296720; doi:10.1371/journal.pone.0032619)
Supplement: Table S2 — Adjusted mean blood based measures by NS-SEC (5 class) and main ethnic group. (DOCX) [file pone.0032619.s002.docx]

**Table S2. Adjusted mean blood based measures by NS-SEC (5 class) and main ethnic group**

| **Outcome & NS-SEC** | **White European (n=1158)** | | **Black African-Caribbean (n=1201)** | | **South Asian (n=1314)** | | **All CHASE (n=4804)** | | **Difference between WE, AC & SA groups** |
| --- | --- | --- | --- | --- | --- | --- | --- | --- | --- |
|  | **mean (95% CI)** | ***P*-value** | **mean (95% CI)** | ***P*-value** | **mean (95% CI)** | ***P*-value** | **mean (95% CI)** | ***P*-value** | ***P*-value§** |
| **HbA1c (%)** |  |  |  |  |  |  |  |  |  |
| Managerial & professional | 5.16 (5.13, 5.20) |  | 5.28 (5.24, 5.31) |  | 5.27 (5.23, 5.31) |  | 5.23 (5.21, 5.25) |  |  |
| Intermediate | 5.19 (5.14, 5.24) |  | 5.21 (5.16, 5.26) |  | 5.32 (5.26, 5.37) |  | 5.24 (5.21, 5.27) |  |  |
| Small employers & own account | 5.18 (5.13, 5.23) |  | 5.29 (5.23, 5.36) |  | 5.29 (5.24, 5.34) |  | 5.25 (5.23, 5.28) |  |  |
| Lower supervisory & technical | 5.20 (5.13, 5.28) |  | 5.32 (5.22, 5.42) |  | 5.33 (5.23, 5.44) |  | 5.28 (5.23, 5.33) |  |  |
| Semi-routine & routine | 5.18 (5.13, 5.22) |  | 5.28 (5.24, 5.32) |  | 5.28 (5.24, 5.31) |  | 5.23 (5.21, 5.25) |  |  |
| Economically inactive | 5.19 (5.14, 5.24) |  | 5.28 (5.23, 5.33) |  | 5.25 (5.21, 5.29) |  | 5.24 (5.21, 5.26) |  |  |
| Unclassified | 5.17 (5.06, 5.28) |  | 5.23 (5.17, 5.29) |  | 5.24 (5.15, 5.33) |  | 5.22 (5.17, 5.26) |  |  |
| p-value NS-SEC (nominal)* |  | 0.87 |  | 0.22 |  | 0.34 |  | 0.35 | 0.30 |
| **Glucose (mmol/L)** |  |  |  |  |  |  |  |  |  |
| Managerial & professional | 4.49 (4.45, 4.53) |  | 4.47 (4.43, 4.51) |  | 4.52 (4.48, 4.57) |  | 4.50 (4.47, 4.52) |  |  |
| Intermediate | 4.51 (4.46, 4.57) |  | 4.50 (4.44, 4.55) |  | 4.55 (4.49, 4.61) |  | 4.52 (4.49, 4.55) |  |  |
| Small employers & own account | 4.53 (4.48, 4.59) |  | 4.52 (4.45, 4.59) |  | 4.58 (4.53, 4.64) |  | 4.54 (4.51, 4.57) |  |  |
| Lower supervisory & technical | 4.56 (4.47, 4.64) |  | 4.47 (4.37, 4.58) |  | 4.54 (4.43, 4.65) |  | 4.52 (4.47, 4.57) |  |  |
| Semi-routine & routine | 4.51 (4.46, 4.55) |  | 4.47 (4.43, 4.51) |  | 4.56 (4.52, 4.60) |  | 4.51 (4.49, 4.54) |  |  |
| Economically inactive | 4.51 (4.46, 4.57) |  | 4.56 (4.50, 4.61) |  | 4.54 (4.50, 4.59) |  | 4.53 (4.51, 4.56) |  |  |
| Unclassified | 4.48 (4.36, 4.60) |  | 4.51 (4.44, 4.57) |  | 4.49 (4.40, 4.59) |  | 4.52 (4.48, 4.57) |  |  |
| p-value NS-SEC (nominal)* |  | 0.69 |  | 0.08 |  | 0.60 |  | 0.11 | 0.63 |
| **Insulin resistance (HOMA-IR)** |  |  |  |  |  |  |  |  |  |
| Managerial & professional | 0.72 (0.68, 0.77) |  | 0.98 (0.92, 1.05) |  | 0.99 (0.92, 1.07) |  | 0.90 (0.86, 0.94) |  |  |
| Intermediate | 0.75 (0.68, 0.83) |  | 0.97 (0.88, 1.07) |  | 0.96 (0.86, 1.06) |  | 0.90 (0.85, 0.95) |  |  |
| Small employers & own account | 0.82 (0.75, 0.90) |  | 1.03 (0.91, 1.17) |  | 1.08 (0.98, 1.19) |  | 0.95 (0.90, 1.01) |  |  |
| Lower supervisory & technical | 0.74 (0.64, 0.87) |  | 1.01 (0.84, 1.22) |  | 1.18 (0.97, 1.43) |  | 0.95 (0.87, 1.05) |  |  |
| Semi-routine & routine | 0.80 (0.74, 0.87) |  | 0.97 (0.89, 1.05) |  | 1.01 (0.95, 1.09) |  | 0.92 (0.88, 0.96) |  |  |
| Economically inactive | 0.87 (0.79, 0.96) |  | 0.88 (0.80, 0.96) |  | 1.00 (0.92, 1.08) |  | 0.91 (0.87, 0.95) |  |  |
| Unclassified | 0.66 (0.53, 0.82) |  | 0.97 (0.86, 1.10) |  | 1.10 (0.93, 1.31) |  | 0.93 (0.86, 1.01) |  |  |
| p-value NS-SEC (nominal)* |  | 0.02 |  | 0.32 |  | 0.29 |  | 0.44 | 0.05 |
| **Triglyceride (mmol/L)** |  |  |  |  |  |  |  |  |  |
| Managerial & professional | 0.75 (0.72, 0.78) |  | 0.71 (0.68, 0.74) |  | 0.88 (0.84, 0.93) |  | 0.79 (0.77, 0.81) |  |  |
| Intermediate | 0.75 (0.70, 0.79) |  | 0.72 (0.68, 0.77) |  | 0.89 (0.83, 0.94) |  | 0.79 (0.76, 0.81) |  |  |
| Small employers & own account | 0.86 (0.81, 0.91) |  | 0.73 (0.67, 0.79) |  | 0.98 (0.92, 1.04) |  | 0.85 (0.82, 0.88) |  |  |
| Lower supervisory & technical | 0.79 (0.72, 0.87) |  | 0.71 (0.63, 0.79) |  | 0.90 (0.80, 1.02) |  | 0.80 (0.76, 0.85) |  |  |
| Semi-routine & routine | 0.83 (0.79, 0.88) |  | 0.71 (0.68, 0.75) |  | 0.86 (0.83, 0.90) |  | 0.80 (0.78, 0.82) |  |  |
| Economically inactive | 0.81 (0.77, 0.86) |  | 0.71 (0.67, 0.75) |  | 0.91 (0.87, 0.96) |  | 0.82 (0.79, 0.84) |  |  |
| Unclassified | 0.79 (0.69, 0.90) |  | 0.69 (0.64, 0.74) |  | 0.98 (0.89, 1.09) |  | 0.82 (0.78, 0.86) |  |  |
| p-value NS-SEC (nominal)* |  | <0.001 |  | 0.99 |  | 0.01 |  | 0.004 | 0.07 |
| **HDL (mmol/L)** |  |  |  |  |  |  |  |  |  |
| Managerial & professional | 1.51 (1.48, 1.54) |  | 1.51 (1.48, 1.54) |  | 1.46 (1.43, 1.50) |  | 1.50 (1.48, 1.52) |  |  |
| Intermediate | 1.49 (1.44, 1.54) |  | 1.54 (1.49, 1.59) |  | 1.48 (1.44, 1.53) |  | 1.50 (1.47, 1.52) |  |  |
| Small employers & own account | 1.48 (1.43, 1.52) |  | 1.52 (1.46, 1.58) |  | 1.40 (1.35, 1.44) |  | 1.47 (1.45, 1.50) |  |  |
| Lower supervisory & technical | 1.57 (1.49, 1.64) |  | 1.45 (1.36, 1.54) |  | 1.44 (1.36, 1.53) |  | 1.49 (1.45, 1.53) |  |  |
| Semi-routine & routine | 1.47 (1.43, 1.51) |  | 1.50 (1.46, 1.54) |  | 1.45 (1.42, 1.48) |  | 1.48 (1.46, 1.49) |  |  |
| Economically inactive | 1.45 (1.41, 1.50) |  | 1.52 (1.47, 1.56) |  | 1.43 (1.40, 1.47) |  | 1.46 (1.44, 1.48) |  |  |
| Unclassified | 1.52 (1.41, 1.63) |  | 1.53 (1.47, 1.59) |  | 1.43 (1.35, 1.51) |  | 1.48 (1.44, 1.51) |  |  |
| p-value NS-SEC (nominal)* |  | 0.08 |  | 0.64 |  | 0.10 |  | 0.03 | 0.18 |
| **CRP (mg/L)** |  |  |  |  |  |  |  |  |  |
| Managerial & professional | 0.39 (0.34, 0.45) |  | 0.51 (0.45, 0.59) |  | 0.56 (0.47, 0.66) |  | 0.48 (0.45, 0.52) |  |  |
| Intermediate | 0.36 (0.29, 0.45) |  | 0.57 (0.46, 0.70) |  | 0.64 (0.52, 0.79) |  | 0.53 (0.47, 0.59) |  |  |
| Small employers & own account | 0.42 (0.34, 0.51) |  | 0.51 (0.39, 0.66) |  | 0.67 (0.55, 0.82) |  | 0.52 (0.46, 0.58) |  |  |
| Lower supervisory & technical | 0.44 (0.32, 0.61) |  | 0.81 (0.54, 1.20) |  | 0.84 (0.56, 1.28) |  | 0.59 (0.49, 0.72) |  |  |
| Semi-routine & routine | 0.42 (0.35, 0.50) |  | 0.56 (0.47, 0.66) |  | 0.60 (0.52, 0.68) |  | 0.52 (0.48, 0.56) |  |  |
| Economically inactive | 0.46 (0.38, 0.56) |  | 0.42 (0.35, 0.51) |  | 0.55 (0.46, 0.65) |  | 0.49 (0.44, 0.54) |  |  |
| Unclassified | 0.47 (0.30, 0.76) |  | 0.46 (0.36, 0.60) |  | 0.55 (0.38, 0.79) |  | 0.47 (0.39, 0.55) |  |  |
| p-value NS-SEC (nominal)* |  | 0.63 |  | 0.05 |  | 0.28 |  | 0.34 | 0.33 |

Mean: adjusted for sex, age, month and school (random effect). Missing values: age (n=1), glucose (n=33), insulin resistance (n=151), CRP (n=159).

95% CI: 95% confidence interval for the mean

¶ Adjusted for ethnicity (all groups included)

§interaction test of NS-SEC and main ethnic groups (white European, black African-Caribbean, South Asian) and excluding "unclassified" NS-SEC group fitting NS-SEC as a categorical variable

*p-value for NS-SEC fitted as an unordered nominal variable (excluding unclassified group)
