# Supplementary material for: Socio-Economic Position and Type 2 Diabetes Risk Factors: Patterns in UK Children of South Asian, Black African-Caribbean and White European Origin
Source: PLoS One. 2012 Mar 7;7(3):e32619. doi: 10.1371/journal.pone.0032619 (PMC3296720; doi:10.1371/journal.pone.0032619)
Supplement: Table S3 — Adjusted mean physical measures by NS-SEC and ethnic sub-group. (DOCX) [file pone.0032619.s003.docx]

**Table S3. Adjusted mean physical measures by NS-SEC and ethnic sub-group**

|  | **Black Caribbean (n=461)** | | **Black African (n=656)** | | **Difference between black Caribbean & black African groups** | | **Indian (n=408)** | | **Pakistani (n=477)** | | **Bangladeshi (n=323)** | | **Difference between Indian, Pakistani & Bangladeshi groups** | |
| --- | --- | --- | --- | --- | --- | --- | --- | --- | --- | --- | --- | --- | --- | --- |
| **Outcome & NS-SEC** | **Mean (95% CI)** | ***P*-value** | **Mean (95% CI)** | ***P*-value** | ***P*-value§** | ***P*-value‡** | **Mean (95% CI)** | ***P*-value** | **Mean (95% CI)** | ***P*-value** | **Mean (95% CI)** | **P-value** | ***P*-value§** | ***P*-value‡** |
| **Height (cm)** |  |  |  |  |  |  |  |  |  |  |  |  |  |  |
| Managerial & professional | 143.3 (142.2, 144.4) |  | 143.5 (142.6, 144.5) |  |  |  | 138.2 (137.0, 139.4) |  | 139.8 (138.4, 141.2) |  | 136.3 (134.3, 138.4) |  |  |  |
| Intermediate | 142.6 (141.5, 143.8) |  | 142.7 (141.5, 144.0) |  |  |  | 138.4 (137.3, 139.5) |  | 139.4 (138.3, 140.6) |  | 137.3 (135.3, 139.2) |  |  |  |
| Routine & manual | 142.7 (141.4, 143.9) |  | 143.0 (141.9, 144.0) |  |  |  | 139.4 (138.1, 140.6) |  | 138.6 (137.6, 139.7) |  | 138.3 (137.2, 139.5) |  |  |  |
| Economically inactive | 141.8 (139.6, 144.0) |  | 141.5 (140.4, 142.6) |  |  |  | 136.9 (134.6, 139.3) |  | 138.8 (137.5, 140.0) |  | 138.2 (136.9, 139.4) |  |  |  |
| Unclassified | 142.9 (140.5, 145.3) |  | 144.7 (143.1, 146.4) |  |  |  | 140.6 (137.4, 143.8) |  | 138.8 (136.2, 141.5) |  | 137.3 (133.8, 140.9) |  |  |  |
| % difference per NS-SEC† | -0.29 (-0.76, 0.19) | 0.22 | -0.41 (-0.74, -0.08) | 0.01 | 0.67 |  | 0.09 (-0.43, 0.60) | 0.74 | -0.28 (-0.71, 0.15) | 0.19 | 0.42 (-0.13, 0.97) | 0.13 | 0.13 |  |
| *P*-value NS-SEC* |  | 0.63 |  | 0.05 |  | 0.43 |  | 0.27 |  | 0.52 |  | 0.36 |  | 0.23 |
| **Weight (kg)** |  |  |  |  |  |  |  |  |  |  |  |  |  |  |
| Managerial & professional | 39.5 (38.1, 41.0) |  | 39.4 (38.1, 40.7) |  |  |  | 33.5 (32.2, 34.9) |  | 34.1 (32.4, 35.8) |  | 34.1 (31.6, 36.8) |  |  |  |
| Intermediate | 39.4 (37.9, 41.0) |  | 38.9 (37.2, 40.7) |  |  |  | 33.4 (32.1, 34.8) |  | 35.4 (34.0, 36.9) |  | 34.7 (32.3, 37.2) |  |  |  |
| Routine & manual | 39.6 (37.9, 41.3) |  | 38.8 (37.4, 40.2) |  |  |  | 35.1 (33.6, 36.7) |  | 34.5 (33.2, 35.8) |  | 35.4 (34.0, 36.9) |  |  |  |
| Economically inactive | 37.0 (34.3, 39.9) |  | 35.7 (34.4, 37.2) |  |  |  | 32.8 (30.1, 35.7) |  | 34.4 (32.8, 36.0) |  | 33.9 (32.4, 35.4) |  |  |  |
| Unclassified | 37.8 (34.8, 41.1) |  | 39.3 (37.2, 41.6) |  |  |  | 35.2 (31.5, 39.5) |  | 34.6 (31.5, 38.0) |  | 34.5 (30.4, 39.1) |  |  |  |
| % difference per NS-SEC† | -1.06 (-3.32, 1.26) | 0.36 | -2.72 (-4.30, -1.12) | 0.001 | 0.23 |  | 0.94 (-1.57, 3.50) | 0.46 | -0.11 (-2.19, 2.00) | 0.91 | -0.45 (-3.11, 2.28) | 0.74 | 0.72 |  |
| *P*-value NS-SEC* |  | 0.45 |  | 0.001 |  | 0.007 |  | 0.29 |  | 0.61 |  | 0.50 |  | 0.67 |
| **Ponderal index (kg/m^3^)** |  |  |  |  |  |  |  |  |  |  |  |  |  |  |
| Managerial & professional | 13.4 (13.1, 13.8) |  | 13.3 (13.0, 13.6) |  |  |  | 12.7 (12.3, 13.1) |  | 12.5 (12.0, 12.9) |  | 13.5 (12.8, 14.2) |  |  |  |
| Intermediate | 13.6 (13.2, 14.0) |  | 13.4 (13.0, 13.8) |  |  |  | 12.6 (12.3, 13.0) |  | 13.1 (12.7, 13.4) |  | 13.4 (12.8, 14.1) |  |  |  |
| Routine & manual | 13.6 (13.2, 14.1) |  | 13.3 (12.9, 13.6) |  |  |  | 13.0 (12.6, 13.4) |  | 12.9 (12.6, 13.3) |  | 13.4 (13.0, 13.8) |  |  |  |
| Economically inactive | 13.0 (12.3, 13.7) |  | 12.6 (12.3, 13.0) |  |  |  | 12.8 (12.0, 13.6) |  | 12.8 (12.4, 13.3) |  | 12.8 (12.4, 13.2) |  |  |  |
| Unclassified | 13.0 (12.2, 13.8) |  | 13.0 (12.5, 13.5) |  |  |  | 12.7 (11.7, 13.8) |  | 12.9 (12.1, 13.8) |  | 13.3 (12.2, 14.6) |  |  |  |
| % difference per NS-SEC† | -0.22 (-1.85, 1.44) | 0.79 | -1.51 (-2.66, -0.35) | 0.01 | 0.20 |  | 0.68 (-1.10, 2.50) | 0.45 | 0.70 (-0.80, 2.22) | 0.35 | -1.73 (-3.61, 0.19) | 0.07 | 0.10 |  |
| *P*-value NS-SEC* |  | 0.43 |  | 0.01 |  | 0.001 |  | 0.62 |  | 0.24 |  | 0.18 |  | 0.11 |
| **Sum of skinfolds (mm)** |  |  |  |  |  |  |  |  |  |  |  |  |  |  |
| Managerial & professional | 40.4 (37.4, 43.7) |  | 39.3 (36.7, 42.1) |  |  |  | 40.8 (37.4, 44.5) |  | 41.0 (37.1, 45.5) |  | 45.3 (38.7, 53.0) |  |  |  |
| Intermediate | 40.9 (37.7, 44.4) |  | 40.1 (36.6, 44.1) |  |  |  | 42.0 (38.7, 45.6) |  | 46.4 (42.7, 50.6) |  | 42.9 (37.0, 49.6) |  |  |  |
| Routine & manual | 40.5 (37.0, 44.2) |  | 42.4 (39.3, 45.8) |  |  |  | 43.8 (39.9, 48.0) |  | 43.5 (40.2, 47.2) |  | 44.6 (40.9, 48.5) |  |  |  |
| Economically inactive | 36.3 (30.9, 42.6) |  | 40.1 (37.0, 43.5) |  |  |  | 39.2 (32.9, 46.7) |  | 41.7 (37.9, 45.9) |  | 40.1 (36.5, 44.0) |  |  |  |
| Unclassified | 35.9 (30.2, 42.8) |  | 40.2 (35.8, 45.1) |  |  |  | 41.9 (33.1, 53.0) |  | 42.3 (34.7, 51.4) |  | 43.8 (33.7, 57.0) |  |  |  |
| % difference per NS-SEC† | -1.78 (-6.40, 3.06) | 0.45 | 1.32 (-2.08, 4.84) | 0.44 | 0.29 |  | 1.16 (-4.01, 6.61) | 0.66 | -0.48 (-4.75, 3.97) | 0.83 | -3.71 (-8.99, 1.88) | 0.18 | 0.43 |  |
| *P*-value NS-SEC* |  | 0.61 |  | 0.52 |  | 0.21 |  | 0.61 |  | 0.22 |  | 0.34 |  | 0.11 |
| **Fat mass index (kg/m^5^)** |  |  |  |  |  |  |  |  |  |  |  |  |  |  |
| Managerial & professional | 1.81 (1.66, 1.97) |  | 1.83 (1.70, 1.97) |  |  |  | 1.75 (1.59, 1.93) |  | 1.67 (1.50, 1.86) |  | 2.10 (1.78, 2.48) |  |  |  |
| Intermediate | 1.84 (1.69, 2.01) |  | 1.95 (1.76, 2.15) |  |  |  | 1.74 (1.59, 1.91) |  | 1.96 (1.79, 2.15) |  | 1.99 (1.70, 2.33) |  |  |  |
| Routine & manual | 1.89 (1.71, 2.08) |  | 1.88 (1.73, 2.04) |  |  |  | 1.84 (1.66, 2.03) |  | 1.91 (1.75, 2.08) |  | 1.99 (1.81, 2.18) |  |  |  |
| Economically inactive | 1.55 (1.31, 1.83) |  | 1.67 (1.53, 1.83) |  |  |  | 1.66 (1.37, 2.03) |  | 1.74 (1.57, 1.93) |  | 1.89 (1.71, 2.09) |  |  |  |
| Unclassified | 1.67 (1.38, 2.01) |  | 1.75 (1.55, 1.98) |  |  |  | 1.73 (1.34, 2.22) |  | 1.70 (1.38, 2.10) |  | 2.00 (1.51, 2.65) |  |  |  |
| % difference per NS-SEC† | -1.58 (-6.57, 3.67) | 0.54 | -2.45 (-5.95, 1.19) | 0.18 | 0.78 |  | 0.68 (-4.96, 6.64) | 0.82 | 0.55 (-4.06, 5.38) | 0.81 | -3.16 (-8.85, 2.89) | 0.29 | 0.56 |  |
| *P*-value NS-SEC* |  | 0.24 |  | 0.11 |  | 0.03 |  | 0.78 |  | 0.08 |  | 0.72 |  | 0.21 |
| **Waist circumference (cm)** |  |  |  |  |  |  |  |  |  |  |  |  |  |  |
| Managerial & professional | 64.8 (63.3, 66.3) |  | 64.3 (63.0, 65.6) |  |  |  | 62.2 (60.7, 63.8) |  | 61.8 (60.0, 63.7) |  | 64.0 (61.2, 67.0) |  |  |  |
| Intermediate | 64.8 (63.2, 66.3) |  | 64.1 (62.4, 65.9) |  |  |  | 61.7 (60.3, 63.2) |  | 64.6 (63.1, 66.2) |  | 63.4 (60.8, 66.1) |  |  |  |
| Routine & manual | 65.5 (63.8, 67.2) |  | 64.1 (62.7, 65.6) |  |  |  | 63.5 (61.9, 65.3) |  | 64.0 (62.5, 65.5) |  | 64.3 (62.7, 65.9) |  |  |  |
| Economically inactive | 62.8 (59.9, 65.8) |  | 62.6 (61.1, 64.1) |  |  |  | 61.9 (58.8, 65.1) |  | 63.2 (61.4, 64.9) |  | 62.1 (60.4, 63.8) |  |  |  |
| Unclassified | 63.0 (59.9, 66.3) |  | 64.1 (62.0, 66.3) |  |  |  | 62.2 (58.1, 66.6) |  | 63.7 (60.1, 67.4) |  | 63.0 (58.3, 67.9) |  |  |  |
| % difference per NS-SEC† | -0.26 (-1.65, 1.15) | 0.72 | -0.76 (-1.74, 0.23) | 0.12 | 0.55 |  | 0.55 (-0.97, 2.09) | 0.47 | 0.45 (-0.83, 1.73) | 0.49 | -1.00 (-2.61, 0.64) | 0.22 | 0.29 |  |
| *P*-value NS-SEC* |  | 0.48 |  | 0.31 |  | 0.07 |  | 0.43 |  | 0.13 |  | 0.29 |  | 0.27 |

Mean: adjusted for sex, age, observer, month and school (random effect).

P-value: statistical significance of the association for each NS-SEC group relative to baseline (managerial & professional) unless specified otherwise

95% CI: 95% confidence interval

§difference in NSSEC slopes between ethnic sub-groups

‡difference in NSSEC associations between ethnic sub-groups

†from professional to economically inactive (excluding unclassified group)

*p-value for NS-SEC fitted as an unordered nominal variable (excluding unclassified group)
