# Supplementary material for: Socio-Economic Position and Type 2 Diabetes Risk Factors: Patterns in UK Children of South Asian, Black African-Caribbean and White European Origin
Source: PLoS One. 2012 Mar 7;7(3):e32619. doi: 10.1371/journal.pone.0032619 (PMC3296720; doi:10.1371/journal.pone.0032619)
Supplement: Table S4 — Adjusted mean blood based measures by NS-SEC and ethnic sub-group. (DOCX) [file pone.0032619.s004.docx]

**Table S4. Adjusted mean blood based measures by NS-SEC and ethnic sub-group**

|  | **Caribbean (n=461)** | | **African (n=656)** | | **Difference between Caribbean & African** | | **Indian (n=408)** | | **Pakistani (n=477)** | | **Bangladeshi (n=323)** | | **Difference between Indian, Pakistani & Bangladeshi** | |
| --- | --- | --- | --- | --- | --- | --- | --- | --- | --- | --- | --- | --- | --- | --- |
| **Outcome & NS-SEC** | **Mean (95% CI)** | ***P*-value** | **Mean (95% CI)** | ***P*-value** | ***P*-value§** | ***P*-value‡** | **Mean (95% CI)** | ***P*-value** | **Mean (95% CI)** | ***P*-value** | **Mean (95% CI)** | **P-value** | ***P*-value§** | ***P*-value‡** |
| **HbA1c (%)** |  |  |  |  |  |  |  |  |  |  |  |  |  |  |
| Managerial & professional | 5.30 (5.25, 5.36) |  | 5.28 (5.23, 5.33) |  |  |  | 5.29 (5.23, 5.35) |  | 5.32 (5.25, 5.39) |  | 5.18 (5.07, 5.28) |  |  |  |
| Intermediate | 5.22 (5.16, 5.27) |  | 5.27 (5.21, 5.34) |  |  |  | 5.31 (5.26, 5.37) |  | 5.32 (5.26, 5.38) |  | 5.27 (5.17, 5.36) |  |  |  |
| Routine & manual | 5.29 (5.23, 5.35) |  | 5.29 (5.24, 5.35) |  |  |  | 5.30 (5.23, 5.36) |  | 5.33 (5.27, 5.39) |  | 5.22 (5.16, 5.28) |  |  |  |
| Economically inactive | 5.12 (5.01, 5.22) |  | 5.33 (5.27, 5.38) |  |  |  | 5.37 (5.25, 5.49) |  | 5.27 (5.20, 5.33) |  | 5.20 (5.14, 5.26) |  |  |  |
| Unclassified | 5.27 (5.15, 5.38) |  | 5.21 (5.14, 5.29) |  |  |  | 5.34 (5.18, 5.50) |  | 5.16 (5.03, 5.29) |  | 5.26 (5.08, 5.44) |  |  |  |
| % difference per NS-SEC† | -0.64 (-1.24, -0.03) | 0.04 | 0.30 (-0.13, 0.73) | 0.17 | 0.01 |  | 0.29 (-0.37, 0.96) | 0.38 | -0.27 (-0.82, 0.29) | 0.33 | -0.04 (-0.76, 0.68) | 0.90 | 0.43 |  |
| P-value NS-SEC* |  | 0.005 |  | 0.49 |  | 0.01 |  | 0.62 |  | 0.48 |  | 0.59 |  | 0.79 |
| **Glucose (mmol/L)** |  |  |  |  |  |  |  |  |  |  |  |  |  |  |
| Managerial & professional | 4.49 (4.43, 4.54) |  | 4.47 (4.42, 4.52) |  |  |  | 4.51 (4.45, 4.58) |  | 4.52 (4.44, 4.59) |  | 4.60 (4.49, 4.72) |  |  |  |
| Intermediate | 4.53 (4.47, 4.59) |  | 4.51 (4.44, 4.57) |  |  |  | 4.56 (4.50, 4.62) |  | 4.59 (4.52, 4.65) |  | 4.57 (4.47, 4.68) |  |  |  |
| Routine & manual | 4.46 (4.40, 4.53) |  | 4.48 (4.42, 4.53) |  |  |  | 4.55 (4.49, 4.62) |  | 4.58 (4.52, 4.64) |  | 4.59 (4.52, 4.65) |  |  |  |
| Economically inactive | 4.43 (4.32, 4.55) |  | 4.59 (4.53, 4.65) |  |  |  | 4.52 (4.40, 4.65) |  | 4.52 (4.45, 4.59) |  | 4.61 (4.54, 4.69) |  |  |  |
| Unclassified | 4.47 (4.35, 4.60) |  | 4.52 (4.44, 4.60) |  |  |  | 4.50 (4.34, 4.67) |  | 4.51 (4.38, 4.66) |  | 4.46 (4.28, 4.65) |  |  |  |
| % difference per NS-SEC† | -0.34 (-1.09, 0.41) | 0.36 | 0.70 (0.16, 1.24) | 0.01 | 0.02 |  | 0.21 (-0.61, 1.03) | 0.61 | -0.05 (-0.74, 0.64) | 0.88 | 0.17 (-0.71, 1.06) | 0.70 | 0.89 |  |
| P-value NS-SEC* |  | 0.35 |  | 0.01 |  | 0.12 |  | 0.73 |  | 0.23 |  | 0.90 |  | 0.93 |
| **Insulin resistance (HOMA-IR)** |  |  |  |  |  |  |  |  |  |  |  |  |  |  |
| Managerial & professional | 0.97 (0.87, 1.07) |  | 1.00 (0.91, 1.09) |  |  |  | 0.98 (0.87, 1.09) |  | 0.9 (0.8, 1.0) |  | 1.2 (1.0, 1.5) |  |  |  |
| Intermediate | 1.02 (0.91, 1.13) |  | 0.95 (0.85, 1.07) |  |  |  | 0.94 (0.84, 1.05) |  | 1.0 (0.9, 1.1) |  | 1.2 (1.0, 1.5) |  |  |  |
| Routine & manual | 1.06 (0.94, 1.19) |  | 0.93 (0.84, 1.03) |  |  |  | 1.07 (0.95, 1.21) |  | 0.9 (0.8, 1.0) |  | 1.2 (1.1, 1.4) |  |  |  |
| Economically inactive | 0.83 (0.68, 1.02) |  | 0.89 (0.80, 0.99) |  |  |  | 1.02 (0.81, 1.27) |  | 1.0 (0.9, 1.1) |  | 1.1 (0.9, 1.2) |  |  |  |
| Unclassified | 1.01 (0.81, 1.27) |  | 0.94 (0.81, 1.08) |  |  |  | 1.11 (0.81, 1.52) |  | 1.06 (0.82, 1.37) |  | 1.20 (0.86, 1.68) |  |  |  |
| % difference per NS-SEC† | -0.41 (-6.34, 5.89) | 0.89 | -3.51 (-7.61, 0.77) | 0.10 | 0.40 |  | 3.51 (-3.16, 10.64) | 0.30 | 1.01 (-4.48, 6.81) | 0.72 | -5.09 (-11.62, 1.93) | 0.14 | 0.19 |  |
| P-value NS-SEC* |  | 0.19 |  | 0.44 |  | 0.01 |  | 0.42 |  | 0.28 |  | 0.38 |  | 0.11 |
| **Triglyceride (mmol/L)** |  |  |  |  |  |  |  |  |  |  |  |  |  |  |
| Managerial & professional | 0.75 (0.70, 0.80) |  | 0.68 (0.64, 0.72) |  |  |  | 0.86 (0.80, 0.92) |  | 0.87 (0.80, 0.95) |  | 0.90 (0.80, 1.02) |  |  |  |
| Intermediate | 0.75 (0.71, 0.80) |  | 0.70 (0.65, 0.75) |  |  |  | 0.88 (0.82, 0.94) |  | 0.96 (0.90, 1.03) |  | 1.03 (0.91, 1.15) |  |  |  |
| Routine & manual | 0.73 (0.68, 0.78) |  | 0.70 (0.66, 0.74) |  |  |  | 0.87 (0.81, 0.94) |  | 0.83 (0.78, 0.89) |  | 0.95 (0.88, 1.01) |  |  |  |
| Economically inactive | 0.70 (0.62, 0.80) |  | 0.71 (0.67, 0.76) |  |  |  | 0.91 (0.79, 1.04) |  | 0.89 (0.83, 0.96) |  | 0.93 (0.86, 1.00) |  |  |  |
| Unclassified | 0.74 (0.65, 0.85) |  | 0.65 (0.59, 0.71) |  |  |  | 0.97 (0.80, 1.16) |  | 0.88 (0.75, 1.02) |  | 1.26 (1.02, 1.54) |  |  |  |
| % difference per NS-SEC† | -1.72 (-5.34, 2.04) | 0.36 | 1.36 (-1.30, 4.10) | 0.31 | 0.18 |  | 0.92 (-3.12, 5.12) | 0.66 | -1.30 (-4.62, 2.13) | 0.44 | -0.60 (-4.90, 3.89) | 0.79 | 0.70 |  |
| P-value NS-SEC* |  | 0.76 |  | 0.72 |  | 0.35 |  | 0.93 |  | 0.02 |  | 0.43 |  | 0.14 |
| **HDL (mmol/L)** |  |  |  |  |  |  |  |  |  |  |  |  |  |  |
| Managerial & professional | 1.49 (1.44, 1.54) |  | 1.53 (1.48, 1.57) |  |  |  | 1.44 (1.39, 1.49) |  | 1.54 (1.48, 1.61) |  | 1.46 (1.37, 1.56) |  |  |  |
| Intermediate | 1.55 (1.50, 1.61) |  | 1.49 (1.44, 1.55) |  |  |  | 1.49 (1.44, 1.54) |  | 1.41 (1.36, 1.46) |  | 1.38 (1.30, 1.47) |  |  |  |
| Routine & manual | 1.47 (1.41, 1.52) |  | 1.51 (1.46, 1.56) |  |  |  | 1.50 (1.44, 1.56) |  | 1.48 (1.43, 1.53) |  | 1.36 (1.31, 1.41) |  |  |  |
| Economically inactive | 1.56 (1.46, 1.67) |  | 1.49 (1.44, 1.54) |  |  |  | 1.47 (1.36, 1.58) |  | 1.46 (1.41, 1.52) |  | 1.38 (1.33, 1.44) |  |  |  |
| Unclassified | 1.50 (1.39, 1.61) |  | 1.53 (1.46, 1.61) |  |  |  | 1.44 (1.30, 1.59) |  | 1.44 (1.33, 1.56) |  | 1.38 (1.24, 1.54) |  |  |  |
| % difference per NS-SEC† | 0.33 (-1.66, 2.36) | 0.74 | -0.66 (-2.06, 0.76) | 0.35 | 0.42 |  | 1.30 (-0.88, 3.53) | 0.24 | -0.78 (-2.57, 1.05) | 0.39 | -1.33 (-3.62, 1.01) | 0.25 | 0.20 |  |
| P-value NS-SEC* |  | 0.08 |  | 0.71 |  | 0.11 |  | 0.44 |  | 0.01 |  | 0.30 |  | 0.05 |
| **CRP (mg/L)** |  |  |  |  |  |  |  |  |  |  |  |  |  |  |
| Managerial & professional | 0.59 (0.48, 0.74) |  | 0.50 (0.42, 0.60) |  |  |  | 0.54 (0.43, 0.69) |  | 0.56 (0.42, 0.74) |  | 0.62 (0.41, 0.95) |  |  |  |
| Intermediate | 0.61 (0.48, 0.76) |  | 0.53 (0.41, 0.68) |  |  |  | 0.63 (0.50, 0.79) |  | 0.64 (0.51, 0.81) |  | 0.75 (0.50, 1.11) |  |  |  |
| Routine & manual | 0.57 (0.45, 0.73) |  | 0.61 (0.49, 0.75) |  |  |  | 0.59 (0.46, 0.76) |  | 0.69 (0.55, 0.86) |  | 0.55 (0.43, 0.70) |  |  |  |
| Economically inactive | 0.44 (0.28, 0.68) |  | 0.45 (0.36, 0.57) |  |  |  | 0.54 (0.34, 0.87) |  | 0.62 (0.48, 0.80) |  | 0.53 (0.41, 0.69) |  |  |  |
| Unclassified | 0.41 (0.25, 0.66) |  | 0.47 (0.34, 0.65) |  |  |  | 0.48 (0.24, 0.94) |  | 0.59 (0.35, 1.02) |  | 0.56 (0.28, 1.15) |  |  |  |
| % difference per NS-SEC† | -5.88 (-17.68, 7.62) | 0.37 | -0.55 (-9.43, 9.21) | 0.91 | 0.50 |  | 1.64 (-11.92, 17.28) | 0.82 | 3.57 (-8.10, 16.73) | 0.56 | -8.07 (-21.11, 7.13) | 0.27 | 0.45 |  |
| P-value NS-SEC* |  | 0.63 |  | 0.30 |  | 0.41 |  | 0.81 |  | 0.70 |  | 0.51 |  | 0.79 |

Mean: adjusted for sex, age, month and school (random effect).

P-value: statistical significance of the association for each NS-SEC group relative to baseline (managerial & professional) unless specified otherwise

95% CI: 95% confidence interval

§difference in NSSEC slopes between ethnic sub-groups

‡difference in NSSEC associations between ethnic sub-groups

†from professional to economically inactive (excluding unclassified group)

*p-value for NS-SEC fitted as an unordered nominal variable (excluding unclassified group)
