# Supplementary material for: Socio-Economic Position and Type 2 Diabetes Risk Factors: Patterns in UK Children of South Asian, Black African-Caribbean and White European Origin
Source: PLoS One. 2012 Mar 7;7(3):e32619. doi: 10.1371/journal.pone.0032619 (PMC3296720; doi:10.1371/journal.pone.0032619)
Supplement: Table S5 — Ethnic differences in type 2 diabetes risk factors compared to white European children with and without adjustment for NS-SEC. (DOCX) [file pone.0032619.s005.docx]

**Table S5 Ethnic differences in type 2 diabetes risk factors compared to white European children with and without adjustment for NS-SEC (n=4804)**

|  | Adjusted SES | Black African-Caribbean - white European n=1202 | | South Asian – white European n=1314 | |
| --- | --- | --- | --- | --- | --- |
|  |  | % diff | 95% CI | % diff | 95% CI |
| Height (cm) | No | 2.97 | 2.58, 3.37 | -0.25 | -0.63, 0.12 |
|  | Yes | 2.94 | 2.55, 3.34 | -0.21 | -0.59, 0.16 |
| Weight (kg) | No | 10.29 | 8.23, 12.39 | -2.36 | -4.16, -0.52 |
|  | Yes | 10.29 | 8.22, 12.41 | -2.36 | -4.17, -0.51 |
| Ponderal index (kg/m3) | No | 0.97 | -0.39, 2.35 | -1.65 | -2.96, -0.32 |
|  | Yes | 1.06 | -0.30, 2.45 | -1.75 | -3.06, -0.41 |
| Sum of skinfolds (mm) | No | -2.07 | -5.85, 1.87 | 5.41 | 1.36, 9.62 |
|  | Yes | -1.87 | -5.69, 2.11 | 5.11 | 1.05, 9.35 |
| Fat mass index (kg/m5) | No | 4.83 | 0.47, 9.39 | 7.61 | 3.11, 12.31 |
|  | Yes | 5.34 | 0.92, 9.95 | 7.33 | 2.82, 12.04 |
| Waist circumference (cm) | No | 0.39 | -0.75, 1.54 | -1.32 | -2.43, -0.20 |
|  | Yes | 0.46 | -0.69, 1.63 | -1.40 | -2.51, -0.26 |
| HbA1c (%) | No | 1.89 | 1.38, 2.41 | 2.17 | 1.65, 2.70 |
|  | Yes | 1.96 | 1.45, 2.48 | 2.21 | 1.68, 2.73 |
| Glucose (mmol/L) | No | -0.20 | -0.82, 0.43 | 0.81 | 0.18, 1.46 |
|  | Yes | -0.15 | -0.78, 0.49 | 0.79 | 0.15, 1.43 |
| Insulin (mU/L) | No | 21.94 | 15.86, 28.33 | 30.16 | 23.62, 37.03 |
|  | Yes | 22.41 | 16.27, 28.87 | 30.15 | 23.58, 37.07 |
| Insulin resistance (HOMA-IR) | No | 22.19 | 16.15, 28.56 | 29.70 | 23.21, 36.52 |
|  | Yes | 22.65 | 16.54, 29.09 | 29.74 | 23.21, 36.61 |
| Triglyceride (mmol/L) | No | -10.58 | -13.31, -7.76 | 12.82 | 9.35, 16.39 |
|  | Yes | -10.26 | -13.02, -7.41 | 12.70 | 9.22, 16.29 |
| HDL-cholesterol (mmol/L) | No | 1.89 | 0.23, 3.59 | -2.96 | -4.54, -1.35 |
|  | Yes | 1.87 | 0.20, 3.58 | -2.73 | -4.32, -1.11 |
| C-reactive protein (mg/L) | No | 21.85 | 9.32, 35.82 | 43.57 | 28.98, 59.82 |
|  | Yes | 22.99 | 10.25, 37.19 | 43.67 | 28.97, 60.05 |

Percentage differences adjusted for sex, age, observer (physical measurements), month and school (random effect).

NS-SEC adjustment using categories: managerial & professional, intermediate, small employers & own account, lower supervisory & technical, semi-routine & routine, economically inactive, unclassifiable.

Missing values: skinfolds (n=12), fat mass index (n=64), waist circumference (n=1), glucose (n=33), insulin (n=89), insulin resistance (n=151), C-reactive protein (n=159)
