# Supplementary material for: Socio-Economic Position and Type 2 Diabetes Risk Factors: Patterns in UK Children of South Asian, Black African-Caribbean and White European Origin
Source: PLoS One. 2012 Mar 7;7(3):e32619. doi: 10.1371/journal.pone.0032619 (PMC3296720; doi:10.1371/journal.pone.0032619)
Supplement: Table S6 — Ethnic differences (compared to white European) in type 2 diabetes risk factors by NS-SEC group. (DOCX) [file pone.0032619.s006.docx]

**Table S6 Ethnic differences (compared to white European) in type 2 diabetes risk factors by NS-SEC group**

|  | Economically inactive | | Routine & Manual | | Intermediate | | Managerial & Professional | |
| --- | --- | --- | --- | --- | --- | --- | --- | --- |
|  | % diff§ | 95% CI | % diff§ | 95% CI | % diff§ | 95% CI | % diff§ | 95% CI |
| **South Asian - white European** |  |  |  |  |  |  |  |  |
| Ponderal Index | -4.81 | -7.87, -1.65 | -3.21 | -5.62, -0.74 | -0.09 | -2.61, 2.49 | -0.96 | -3.54, 1.69 |
| Sum of skinfolds | -7.36 | -15.76, 1.89 | 1.49 | -5.69, 9.21 | 11.89 | 3.86, 20.55 | 8.27 | 0.22, 16.97 |
| Fat mass index | -1.38 | -10.96, 9.23 | 3.15 | -4.68, 11.63 | 14.10 | 5.30, 23.64 | 8.71 | 0.01, 18.16 |
| Waist circumference | -4.23 | -6.84, -1.55 | -1.64 | -3.72, 0.48 | -0.63 | -2.76, 1.54 | -0.82 | -3.01, 1.43 |
| Insulin Resistance (HOMA-IR) | 12.82 | -0.08, 27.40 | 29.22 | 17.63, 41.96 | 28.24 | 16.54, 41.13 | 36.11 | 23.28, 50.28 |
| Triglyceride | 11.16 | 3.18, 19.75 | 4.76 | -1.09, 10.95 | 15.65 | 9.10, 22.60 | 17.12 | 10.25, 24.42 |
| **Black African-Caribbean - white European** |  |  |  |  |  |  |  |  |
| Ponderal Index | -5.31 | -8.54, -1.97 | -0.59 | -3.26, 2.15 | 3.91 | 1.13, 6.76 | 3.34 | 0.87, 5.87 |
| Sum of skinfolds | -12.34 | -20.77, -3.03 | -4.54 | -11.81, 3.34 | 1.71 | -6.01, 10.07 | 1.29 | -5.62, 8.70 |
| Fat mass index | -8.80 | -18.13, 1.59 | 0.42 | -7.77, 9.35 | 13.47 | 4.25, 23.49 | 9.57 | 1.55, 18.22 |
| Waist circumference | -3.64 | -6.43, -0.76 | -0.32 | -2.59, 2.01 | 1.26 | -1.04, 3.62 | 2.16 | 0.09, 4.28 |
| Insulin Resistance (HOMA-IR) | -0.92 | -12.89, 12.70 | 21.73 | 9.95, 34.77 | 23.58 | 11.80, 36.60 | 33.13 | 21.63, 45.71 |
| Triglyceride | -13.34 | -19.91, -6.24 | -14.01 | -19.18, -8.52 | -10.25 | -15.62, -4.55 | -5.39 | -10.47, -0.03 |

§Percentage difference adjusted for sex, age, observer (physical measurements), month, school (random effect)
